# Synthesis of Functionalized Diethyl(pyrrolidin-2-yl)phosphonate and Diethyl(5-oxopyrrolidin-2-yl)phosphonate

**DOI:** 10.3390/molecules26113160

**Published:** 2021-05-25

**Authors:** Iwona E. Głowacka, Anna Hartwich, Iwona Rozpara, Dorota G. Piotrowska

**Affiliations:** Bioorganic Chemistry Laboratory, Faculty of Pharmacy, Medical University of Lodz, 90-151 Lodz, Poland; anna.zdzienicka@umed.lodz.pl (A.H.); iwona.rozpara@umed.lodz.pl (I.R.)

**Keywords:** cycloaddition, isoxazolidines, phosphonates, substituted pyrrolidines

## Abstract

Short and efficient syntheses of functionalized (pyrrolidin-2-yl)phosphonate and (5-oxopyrrolidin-2-yl)phosphonate have been developed. The synthetic strategy involved the diastereospecific 1,3-dipolar cycloaddition of *N*-benzyl-*C*-(diethoxyphosphoryl)nitrone to *cis*-1,4-dihydroxybut-2-ene and dimethyl maleate, respectively. *O*,*O*-Diethyl 3-carbamoyl-4-hydroxy(5-oxopyrrolidin-2-yl)phosphonate was obtained from *O*,*O*-diethyl 2-benzyl-4,5-dimethoxycarbonyl(isoxazolidin-3-yl)phosphonate by hydrogenation and subsequent treatment with ammonia, whereas transformation of *O*,*O*-diethyl 2-benzyl-4,5-dihydroxymethyl(isoxazolidin-3-yl)phosphonate into *O*,*O*-diethyl 3-aminomethyl-4-hydroxy(pyrrolidin-2-yl)phosphonate was accomplished by mesylation followed by hydrogenolysis to undergo intramolecular cyclization and the introduction of amino group via ammonolysis. Stereochemistry of the isoxazolidine cycloadducts, as well as the final functionalized (pyrrolidin-2-yl)- and (5-oxopyrrolidin-2-yl)phosphonates were established based on conformational analyses using vicinal H–H, H–P, and C–P couplings and supported by the observed diagnostic NOESY correlation signals.

## 1. Introduction

**Figure 1:**
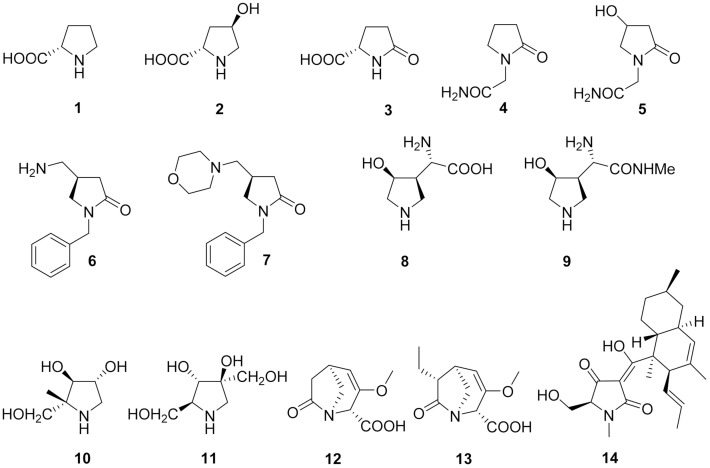
Examples of pyrrolidine- and pyrrolidone-containing biologically active compounds.

Pyrrolidine is an important fragment of many natural products [1,2,3,4] that can be exemplified by complex structures of swainsonin [5], monocotaline [6], lasiocarpine [7], and senecionine [8]. Pyrrolidine and pyrrolidinone moieties are also present in small biologically active molecules. For example, L-proline **1** and its hydoxylated analogue **2** (Figure 1) are the essential components of collagen, accounting for 30% of its composition and playing key roles in the stability of the collagen [9,10].

On the other hand, pyroglutamic acid **3** (Figure 1) is formed as a result of glutamate dehydration [11]. This is an intermediate substrate involved in the glutathione synthesis [12]. For decades, the basic structure of pyroglutamic acid has been modified and resulted in the syntheses of pharmacologically active compounds such as piracetam **4**, oxiracetam **5**, nebracetam **6**, and its morpholine derivative **7** (Figure 1), which belong to “nootropic drugs” used in treatment of CNS diseases such as epilepsy and depression [13,14,15,16,17,18].

Hydroxylated pyrrolidine derivatives **8** and **9** (Figure 1) affected brain Glu levels and at the same time they did not exhibit brain and hepatic toxicity. The only disadvantage of these compounds is their inability to overcome the blood-brain interface [19]. Polyhydroxylated derivative of pyrrolidine **10**, its enantiomer and **11** (Figure 1) have been obtained as inhibitors of α-glucosidases. Moreover, compound **11** demonstrates superior control of blood glucose levels [20]. *On the other hand, antibiotic activity of several* pyrrolidone-containing compounds has been observed, including compounds **12** and **13** [21] containing pyrrolidone ring incorporated in bicyclic system, as well as **14** (derivative of equisetin having additional methyl group at C3 in octahydronaphtalenyl moiety) acting on some multi-drug resistant bacteria [22] (Figure 1), and their resistance to β-lactamase have been recognized.

**Figure 2:**
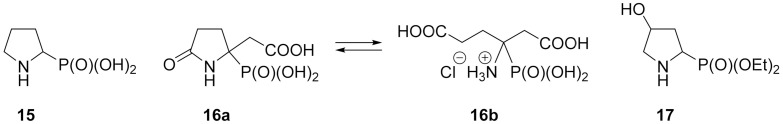
Phosphoproline **15** and its functionalized analogues **16** and **17**.

Over the decades, the importance of phosphonates in medicinal chemistry has been recognized [23,24,25]. Numerous phosphonates have been reported as analogues of biologically important compounds, including inhibitors of several enzymes, as well as antibacterial, antiviral, and fungicidal agents. Phosphonates have also been applied as mimetics of hydroxy- and amino acids in studies on their mode of action in biochemical transformations [26]. For this reason, phosphoproline **15** (Figure 2) [27] and its functionalized analogues received considerable attention and some of them have been successfully incorporated in biologically active systems such as analogues of dipeptides [28,29,30,31,32,33]. On the other hand, pyrrolidinone-containing phosphonate **16** (Figure 2), as a mixture of cyclic and non-cyclic form, has been recognized as inhibit NMCA β-lactamase. Moreover, a good activity of the cyclic form in the mixture of **16a** and **16b** tested against R39 D,D-peptidase has been proved [34].

**Scheme 1:**
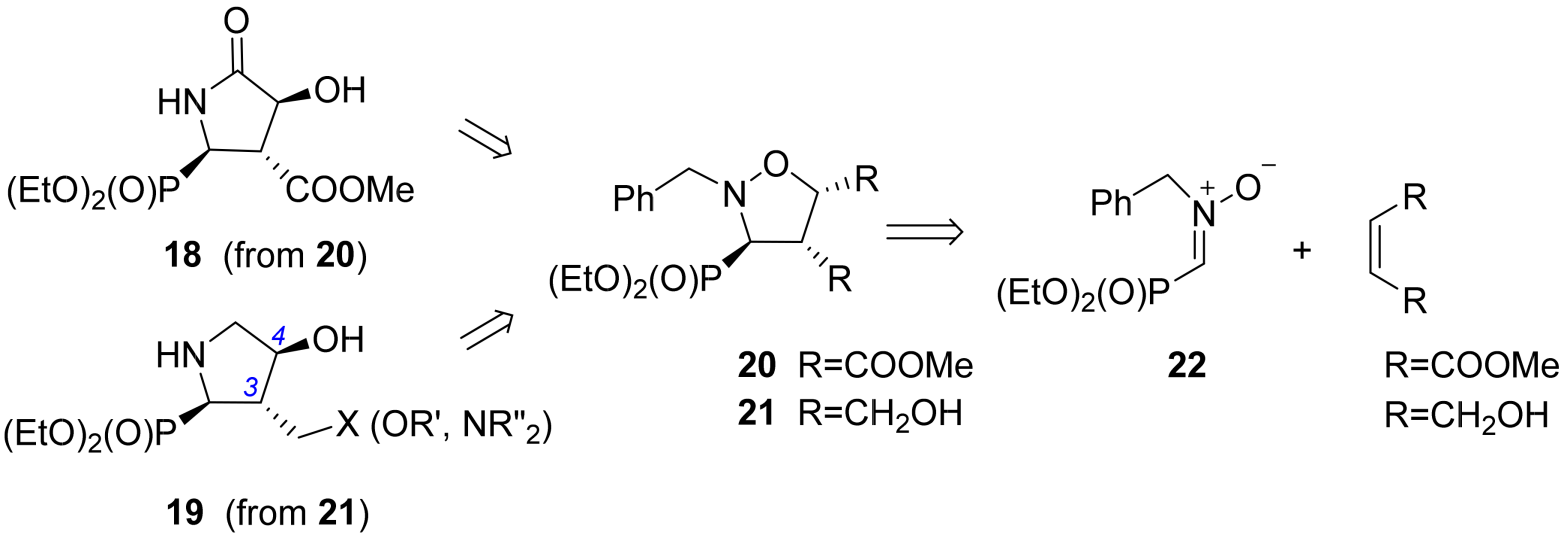
Retrosynthesis of **18** and **19**.

Several years ago, stereoisomers of analogues of proline as respective diethyl phosphonates **17** (Figure 2) hydroxylated at C4 in pyrrolidine ring have been synthesized in our research group [35]. Herein the syntheses of phosphonates **18** and **19** containing pyrrolidine framework functionalized at C3 and C4 are described (Scheme 1). Since *N*-substituted C-phosphorylated nitrones [36] have been successfully applied in the synthesis of various (isoxazolidin-3-yl)phosphonates [36,37] we found them suitable also for the preparation of isoxazolidines **20** and **21**, which could be then transformed into the designed compounds **18** and **19** or their functionalized analogues. While isoxazolidine cycloadducts obtained from allyl alcohol and C-phosphorylated nitrone have already been successfully transformed into compound **17** (Figure 2) having hydroxy group at C4 in pyrrolidine skeleton [35], the application of 1,4-dihydroxybut-2-ene in 1,3-dipolar cycloaddition would allow to synthesis pyrrolidine **19** functionalized in both C3 and C4 positions. On the other hand, rearrangement of isoxazolidine **20** to pyrrolidinone **18** would be possible following the strategy demonstrated for several examples of differently functionalized systems [38,39,40,41].

## 2. Results and Discussion

**Scheme 2:**
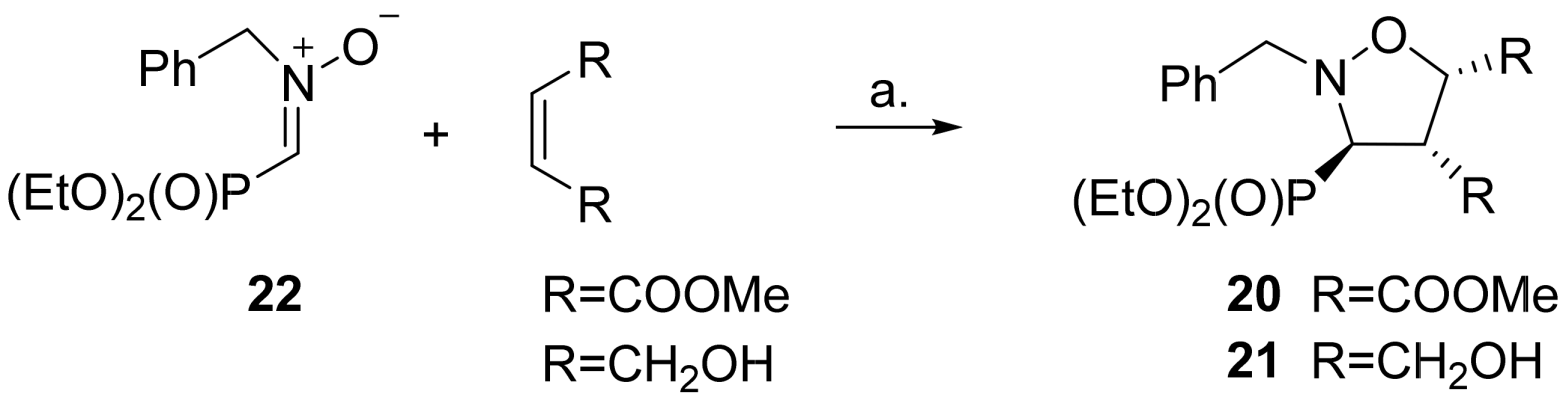
Reaction and conditions: a. toluene, 60 °C (reaction time: 24 h for the synthesis of **20** and 96 h for the synthesis of **21**).

The nitrone **22** was synthesized and fully characterized previously [36]. Cycloaddition of nitrone **22** with dimethyl maleate was then performed and led to the formation of (isoxazolidin-3-yl)phosphonate **20** as a single diastereoisomer in 84% yield after chromatographic purification. Similarly, reaction of nitrone **22** with *cis*-1,4-dihydroxybut-2-ene gave diastereoisomeric cycloadduct **21** in 70% yield after column chromatography (Scheme 2). In both cases, formation of single diastereoisomeric product (**20** or **21**) was proved by the analyses of the ^31^P and ^1^H NMR spectra of the crude product.

**Scheme 3:**
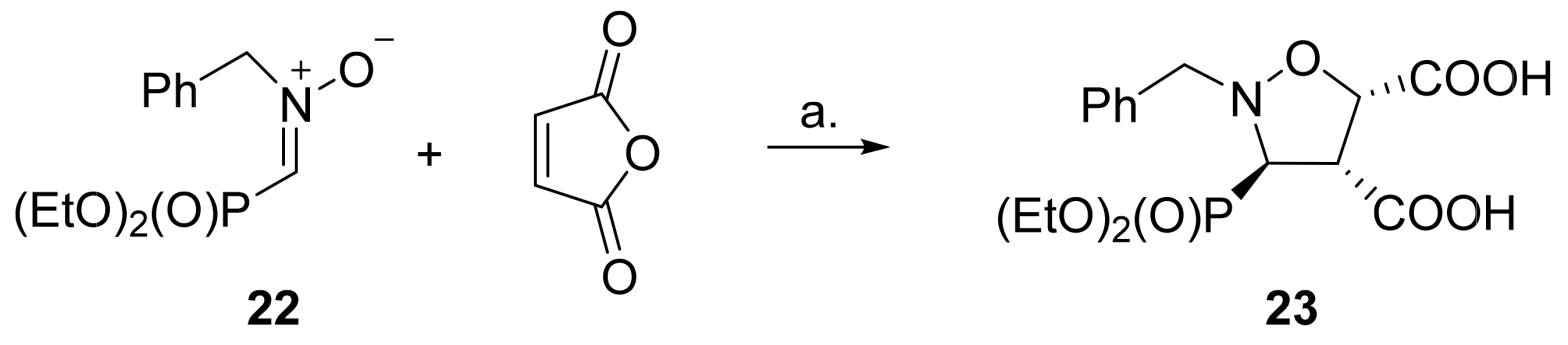
Reaction and conditions: a. toluene, 24 h, 60 °C, 74%.

On the other hand, when maleic anhydride was used in the 1,3-dipolar cycloaddition with nitrone **22** isoxazolidine **23** was obtained exclusively in good yield (Scheme 3).

**Figure 3:**
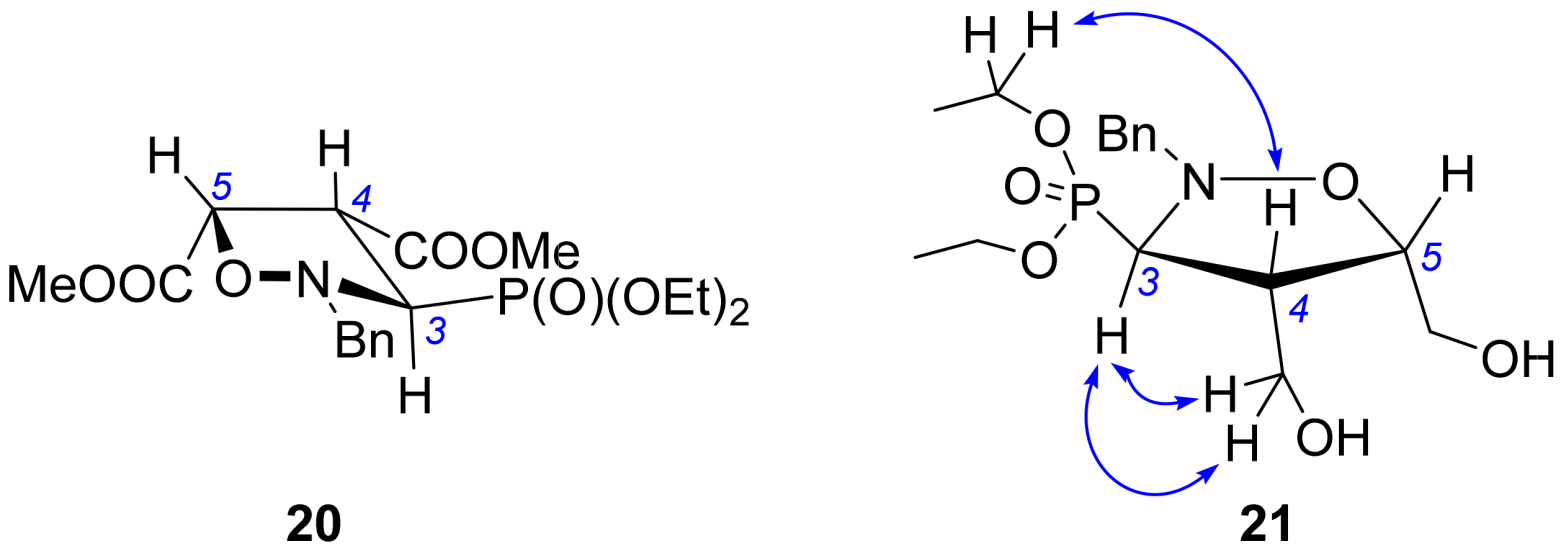
The preferred conformation of **20** and the most important NOESY correlation for phosphonate **21** (blue arrows).

Since *cis*-alkenes were used for cycloadditions (Scheme 2 and Scheme 3), the *cis* relationship between ***H***C4 and ***H***C5 protons in **20** and **21**, as well as in **23** can be arbitrarily assigned. To establish a relative configuration of (isoxazolidin-3-yl)phosphonate **18** the detailed conformational analysis was performed based on *H*CC*P* [42], *H*CC*P* [43,44], and *C*CC*P* [45,46] vicinal constants extracted from the ^1^H and ^13^C-NMR spectra. The vicinal couplings (*J*(*H*-C3C4-*H*) = 8.0 Hz, *J*(*H*-C4C5-*H*) = 8.3 Hz, *J*(*H*-C4C3-*P*) = 16.0 Hz, *J*(*P*-CC-*C5*) = 8.0 Hz, and *J*(*P*-CC-*CO*) = 5.5 Hz) indicate the _3_*E* conformation of isoxazolidine ring. In this conformation the pseudoequatorially located diethoxyphosphoryl group at C3 is in *trans* relationship to both COOMe groups at C4 and C5 positions (Figure 3). On the other hand, to gather evidences for the spatial orientation of substituents in relation to the isoxazolidine moiety in (isoxazolidin-3-yl)phosphonate **21**, NOESY experiment was performed. NOE diagnostic signals between ***H***C4 and C***H***_2_OP as well as between ***H***C3 and C4-C***H***_2_OH protons were noticed (Figure 3), which fully support the *trans* relationship between the *H*C3 and *H*C4 protons.

**Scheme 4:**
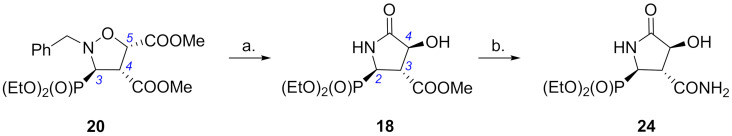
Synthesis of γ-lactam **24**. Reaction and conditions: a. H_2_, Pd(OH)_2_–C, MeOH, rt, 1.01 bar, 24 h, 75% or H_2_, Pd(OH)_2_–C, MeOH, rt, 15 bar, 5 h, 94%; b. aq. NH_3_, MeOH, 17 h, 56%.

Next, transformation of (isoxazolidin-3-yl)phosphonate **20** into (5-oxopyrrolidin-2-yl)phosphonate **24** was performed (Scheme 4). Hydrogenation of the N–O bond together with the removal of benzyl group in isoxazolidine **20** released the free amino group, which subsequently became involved in spontaneous intramolecular cyclization to γ-lactam to produce phosphonate **18** in good yield (75%). When hydrogenation was carried out at a pressure of 15 bar, reaction time was significantly shortened (24 h vs. 5 h); moreover, the application of this procedure allowed to isolate compound **16** in higher yield (94%). Since configurations of all stereogenic centers in isoxazolidine ring (namely at C3, C4, and C5) remain unchanged during this transformation, relative configuration of γ-lactam ring (at C2, C3, and C4, respectively) in **18** can be established unambiguously. 3-Methoxycarbonyl-(5-oxopyrrolidin-2-yl)phosphonate **18** was then successfully transformed into 3-carbamoyl derivative **24** via ammonolysis (56%).

**Figure 4:**
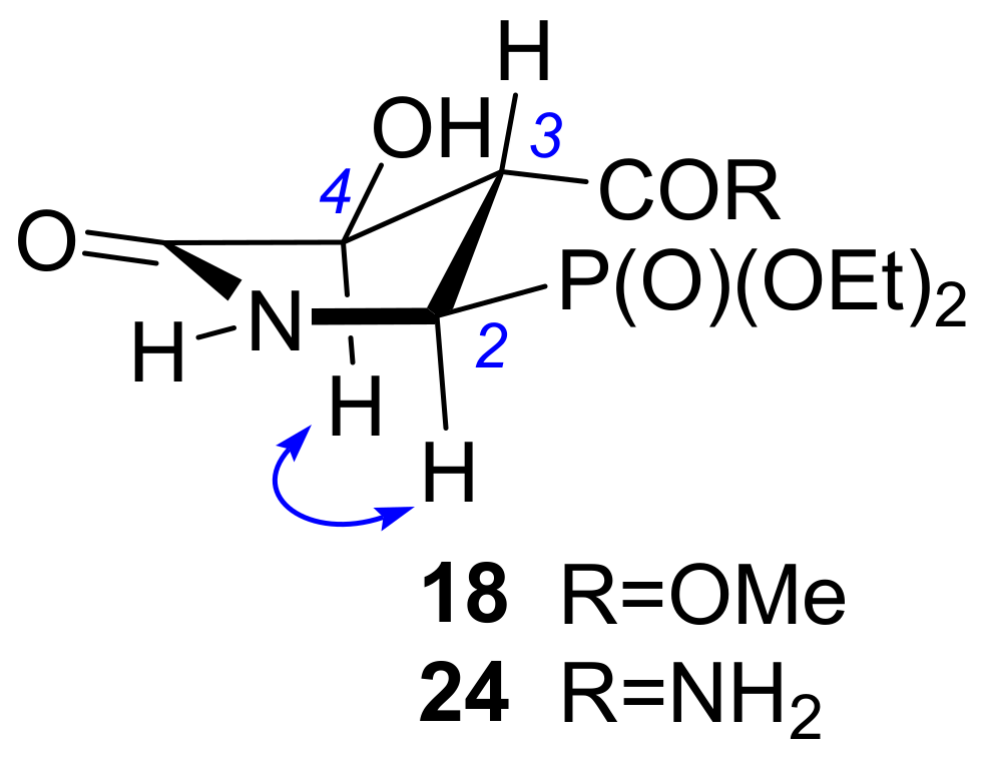
The preferred conformations of **18** and **24** with the most important NOESY correlations (blue arrow).

In order to support the already established relative stereochemistry of (5-oxopyrrolidin-2-yl)phosphonates **18** and **24**, conformational analyses were undertaken. Based on the vicinal couplings found in ^1^H and ^13^C-NMR spectra of **18** (*J*(*H*-C3C4-*H*) = 8.8 Hz, *J*(*H*-C2C3-*H*) = 8.8 Hz, *J*(*H*-C3C2-*P*) = 17.6 Hz, *J*(*P*-CC-*C4*) = 7.8 Hz and *J*(*P*-CN-*CO*) = 7.8 Hz) and **24** (*J*(*H*-C3C4-*H*) = 9.1 Hz, *J*(*H*-C2C3-*H*) = 9.0 Hz, *J*(*H*-C3C2-*P*) = 18.0 Hz, *J*(*P*-CC-*C4*) = 8.7 Hz and *J*(*P*-CN-*CO*) = 7.9 Hz), the preferred ^3^*E* conformation of oxopyrrolidine ring was established in both phosphonates **18** and **24**. In this conformation all substituents at C2, C3, and C4, namely OH, COR, and P(O)(OEt)_2_ groups are located equatorially, consequently hydrogen atoms occupy axial positions (Figure 4). Moreover, when NOESY experiments were performed for both phosphonates **18** and **24**, NOE diagnostic signals between ***H***C4 and ***H***C2 protons were noticed, which fully support their *cis* orientations.

**Scheme 5:**
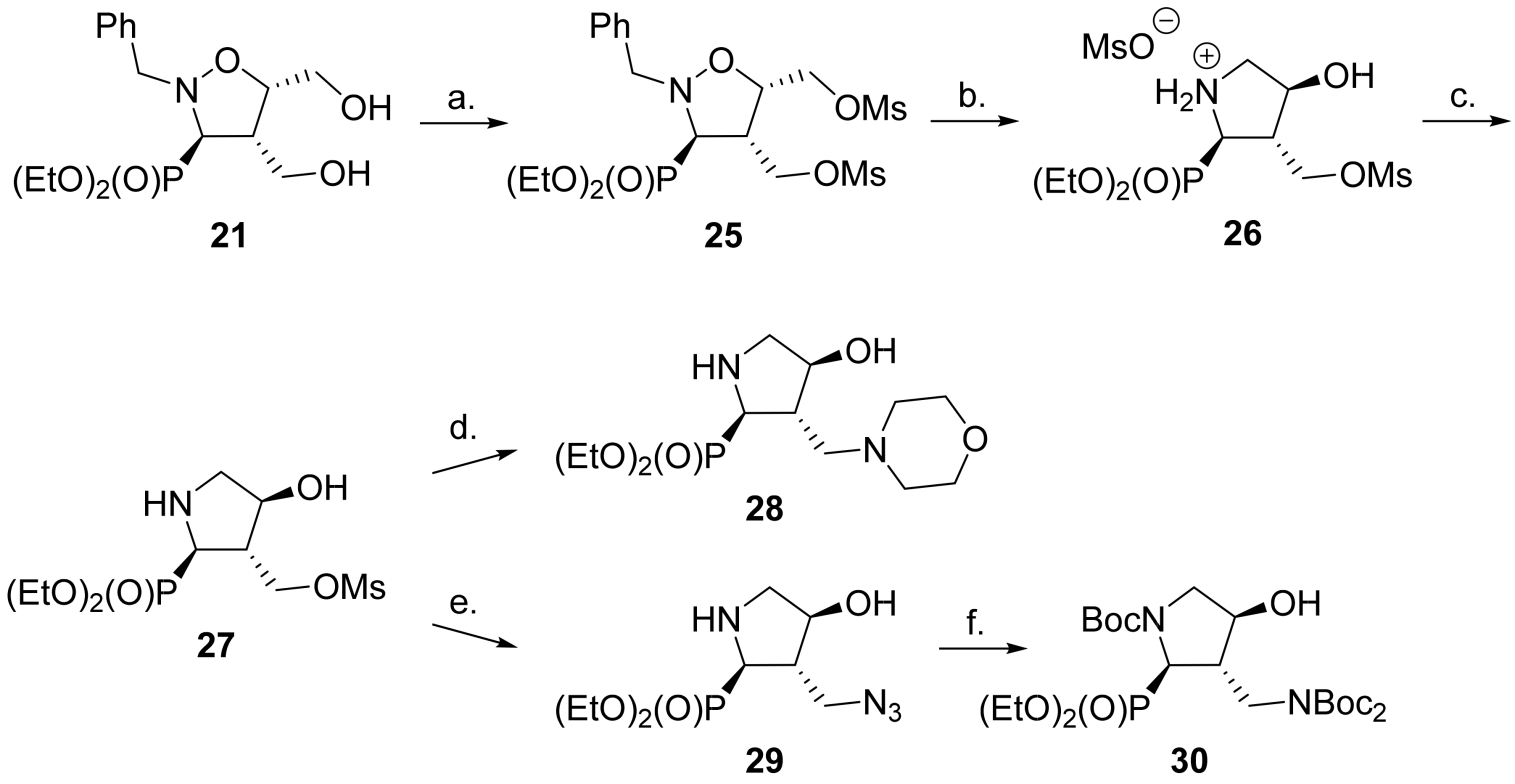
Synthesis of functionalized proline analogues **28** and **30**. Reaction and conditions: a. MsCl, Et_3_N, CH_2_Cl_2_, 0 °C, 2h, 96%; b. H_2_, Pd(OH)_2_–C, MeOH, 1.01 bar, 22 h or H_2_, Pd(OH)_2_–C, MeOH, 15 bar, 6 h; c. K_2_CO_3_, CHCl_3_, rt, 3 h, (38% and 70% in two steps: b and c); d. morpholine, neat, rt, 39 h, 33%; e. NaN_3_, MeOH, 60 °C, 96 h, 43%; f. H_2_, Pd(OH)_2_–C, Boc_2_O, EtOH, rt, 35 h, 69%.

On the other hand, (isoxazolidin-3-yl)phosphonate **21** was found to be a good substrate for the synthesis of functionalized phosphoproline derivative (Scheme 5). Phosphonate **21** was first mesylated to produce *O*,*O*-dimesyl derivative **25**, which was then subjected to hydrogenolysis to accomplish cleavage of N–O bond followed by removal of benzyl group together with intramolecular cyclization to form pyrrolidine ring in compound **26** [35]. The resulted crude ammonium mesylate **26** was then neutralized with potassium carbonate in chloroform to give functionalized phosphoproline analogue **27** (38% yield in two steps). Again, when hydrogenation was carried out at 15 bar pressure, the reaction time required for transformation of (isoxazolidin-3-yl)phosphonate **25** into ammonium mesylate **26** was significantly shortened (22 h vs. 6 h), and after neutralization with potassium carbonate phosphoproline analogue **27** was obtained efficiently (70% yield in two steps). Finally, compound **27** was reacted with morpholine to give **28** in 33% yield. Alternatively, mesyl group in **27** was changed to amino function by treatment with sodium azide followed by hydrogenolysis in the presence of Boc_2_O to produce phosphonate **30** (Scheme 5).

## 3. Materials and Methods

### 3.1. General Information

^1^H-NMR spectra were taken in CDCl_3_, C_6_D_6_ or CD_3_OD on a Bruker Avance III (600 MHz, Bruker Instruments, Karlsruhe, Germany). For spectra recorded in CDCl_3_ and C_6_D_6_ TMS was used as an internal standard; chemical shifts δ are given in ppm with respect to TMS and coupling constants *J* in Hz. ^13^C-NMR and ^31^P-NMR spectra were recorded in a ^1^H-decoupled mode for CDCl_3_, C_6_D_6_ or CD_3_OD solutions on the Bruker Avance III (600 MHz) spectrometer at 151 and 243 MHz, respectively. IR spectral data were measured on a Bruker Alpha-T FT-IR spectrometer (Bruker Optik GmbH, Ettlingen, Germany). Melting points were determined on a Boetius apparatus and are uncorrected. Elemental analyses were performed by the Microanalytical Laboratory of the Faculty of Pharmacy (Medical University of Lodz) on a Perkin Elmer PE 2400 CHNS analyzer (Perkin-Elmer Corp., Norwalk, CT, USA), and their results were found to be in good agreement (±0.3%) with the calculated values. Experiments at 15 bar pressure were carried out in a Büchi pressure reactor (Büchi AG, Uster, Switzerland).

The following adsorbents were used: column chromatography, Merck silica gel 60 (70–230 mesh), analytical TLC, Merck TLC plastic sheets silica gel 60 F_254_. TLC plates were developed in chloroform-methanol solvent systems. Visualization of spots was affected with iodine vapors. All solvents were purified by methods described in the literature.

^1^H-, ^13^C- and ^31^P-NMR spectra of all new synthesized compounds are provided in Supplementary Materials.

### 3.2. General Procedure for the Synthesis of Isoxazolidines ***20***, ***21*** and ***23***

A solution of nitrone **22** (2.0 mmol) and alkene (2.2 mmol) in toluene (4 mL) were stirred at 60 °C for 24 to 96 h (until the disappearance of the nitrone). The reaction mixture was concentrated in vacuo. The crude product was purified by silica gel column.

*Dimethyl 2-benzyl-3-(diethoxyphosphoryl)isoxazolidine-4,5-dicarboxylate (***20***)***.** Compound **20** was prepared from nitrone **22** (2.00 mmol, 0.542 g) and dimethyl maleate (2.20 mmol, 0.276 mL) and purified by column chromatography on a silica gel column with hexane-ethyl acetate (3:2, *v*/*v*) and crystallization (chloroform-hexane). Yield: 84% (0.697 g) as a white solid; m.p. = 75–78 °C. IR (KBr, cm^−1^): ν_max_= 2989, 2953, 2908, 1765, 1738, 1442, 1315, 1269, 1221, 1171, 1057, 1026, 984, 743, 707. ^1^H NMR (600 MHz, CDCl_3_): δ = 7.45–7.42 (m, 2H, *H_Ar_*), 7.34–7.31 (m, 2H, *H_Ar_*), 7.29–7.27 (m, 1H, *H_Ar_*), 4.70 (d, ^3^*J*_H5–H4_ = 8.3 Hz, 1H, *H*C5), 4.52 (d, ^2^*J*_Ha–Hb_=14.0 Hz, 1H, *H*_a_H_b_CPh), 4.28–4.16 (m, 5H, 2 × C*H*_2_OP, H_a_*H*_b_CPh), 3.95 (ddd, ^3^*J*_H4–P_ = 16.3 Hz, ^3^*J*_H4–H5_ = 8.3 Hz,^3^*J*_H4–H3_ = 8,0 Hz, 1H, *H*C4), 3.80 (dd, ^3^*J*_H3–H4_ = 8.0 Hz, ^2^*J*_H3–P_ = 2.8 Hz, 1H, *H*C3), 3.73 (s, 3H, C*H*_3_O), 3.71 (s, 3H, C*H*_3_O), 1.35 (t, ^3^*J* = 7.0 Hz, 3H, C*H*_3_CH_2_OP), 1.31 (t, ^3^*J*= 7.0 Hz, 3H, C*H*_3_CH_2_OP). ^13^C NMR (151 MHz, CDCl_3_): δ = 169.29 (d, ^3^*J*_PCCC_ = 5.5 Hz, *C*(O)C4), 168.88 (*C*(O)C5), 137.16, 129.10, 128.26, 127.41, 77.39 (d, ^3^*J*_PCCC_ = 8.0 Hz, C5), 63.96 (d, ^1^*J*_PC_ = 169.6 Hz, C3) 63.93 (d, ^3^*J* = 6.5 Hz, *C*H_2_N), 62.90 (d, ^2^*J* = 5.7 Hz, *C*OP), 62.88 (d, ^2^*J* = 5.7 Hz, *C*OP), 53.45 (*C*H_3_O), 52.76 (*C*H_3_O), 52.47 (C4), 16.54 (d, ^3^*J* = 5.8 Hz, *C*COP), 16.35 (d, ^3^*J* = 5.7 Hz, *C*COP). ^31^P NMR (243 MHz, CDCl_3_): δ = 19.14. Analysis Calculated for C_18_H_26_NO_8_P: C, 52.05; H, 6.31; N, 3.37; Found: C, 52.05; H, 6.31; N, 3.36.

*Diethyl 2-benzyl-4,5-dihydroxy-isoxazolidinyl-3-phosphonate (***21***)***.** Compound **21** was prepared from nitrone **22** (2.00 mmol, 0.542 g) and *cis*-2-butene-1,4-diol (2.20 mmol, 0.194 mL) and purified by column chromatography on a silica gel column with chloroform-methanol (50:1, *v*/*v*). Yield: 70% (0.502 g) as a yellowish oil. IR (film, cm^−1^): ν_max_= 3385, 3064, 3032, 1497, 1229, 1050, 1025, 973, 740, 573. ^1^H NMR (600 MHz, CDCl_3_): δ = 7.40–7.38 (m, 2H, *H_Ar_*), 7.35–7.30 (m, 2H, *H_Ar_*), 7.30–7.26 (m, 1H, *H_Ar_*), 4.55 (d, ^2^*J*_HA-HB_ = 14.5 Hz, 1H, *H*_A_H_B_CPh), 4.32–4.20 (m, 4H, 2 × C*H*_2_OP), 4.13 (ddd, ^3^*J*_H5–H4_ = 9.7 Hz, ^3^*J*_H5–Ha_ = 5.9 Hz, ^3^*J*_H5–Hb_ = 3.5 Hz, 1H, *H*C5), 3.89 (d, ^2^*J*_HA–HB_ = 14.5 Hz, 1H, H_A_*H*_B_CPh), 3.86 (dd, ^2^*J*_Ha–Hb_ = 12.5 Hz, ^3^*J*_Ha–H5_ = 5.9 Hz, 1H, *H*_a_H_b_C-C5), 3.76 (dd, ^2^*J*_Ha–Hb_ = 12.5 Hz, ^3^*J*_Hb–H5_ = 3.5 Hz, 1H, H_a_*H*_b_C-C5), 3.78–3.74 (m, 2H, C*H*_2_-C4), 3.10–3.00 (m, 2H, *H*C4, *H*C3), 1.38 (t, ^3^*J* = 7.2 Hz, 3H, C*H*_3_CH_2_OP), 1.36 (t, ^3^*J*= 7.2 Hz, 3H, C*H*_3_CH_2_OP). ^1^H NMR (600 MHz, C_6_D_6_): δ = 7.54–7.51 (m, 2H, *H_Ar_*), 7.24–7.21 (m, 2H, *H_Ar_*), 7.14–7.10 (m, 1H, *H_Ar_*), 4.54 (d, ^2^*J*_HA–HB_ = 14.4 Hz, 1H, *H*_A_H_B_CPh), 4.28 (dt, ^3^*J*_H5–H4_ = 6.8 Hz, ^3^*J*_H5–Ha_ = 6.8 Hz, ^3^*J*_H5–Hb_ = 4.2 Hz, 1H, *H*C5), 4.08–3.94 (m, 4H, 2 × C*H*_2_OP), 3.89 (d, ^2^*J*_HA–HB_ = 14.4 Hz, 1H, H_A_*H*_B_CPh), 3.89 (dd, ^2^*J*_Ha–Hb_ = 12.0 Hz, ^3^*J*_Ha–H5_ = 6.8 Hz, 1H, *H*_a_H_b_C-C5), 3.87 (dd, ^2^*J*_Ha–Hb_ = 11.4 Hz, ^3^*J*_Ha–H4_ = 7.9 Hz, 1H, *H*_a_H_b_C-C4), 3.79 (dd, ^2^*J*_Ha–Hb_ = 12.0 Hz, ^3^*J*_Hb–H5_ = 4.2 Hz, 1H, H_a_*H*_b_C-C5), 3.76 (dd, ^2^*J*_Ha–Hb_ = 11.4 Hz, ^3^*J*_Hb–H4_ = 4.0 Hz, 1H, H_a_*H*_b_C-C4), 3.20 (ddddd, ^3^*J*_H4–P_ = 18.7 Hz, ^3^*J*_H4–Ha_ = 7.9 Hz, ^3^*J*_H4–H5_ = 6.8 Hz, ^3^*J*_H4–H3_ = 6.8 Hz, ^3^*J*_H4–Hb_ = 4.0 Hz, 1H, *H*C4), 3.07 (dd, ^3^*J*_H3–H4_ = 6.8 Hz, ^2^*J*_H3–P_ = 1.1 Hz, 1H, *H*C3), 1.06 (t, ^3^*J* = 7.0 Hz, 3H, C*H*_3_CH_2_OP), 1.04 (t, ^3^*J* = 7.0 Hz, 3H, C*H*_3_CH_2_OP). ^13^C NMR (151 MHz, C_6_D_6_): δ = 137.94, 129.02, 128.13, 127.99, 127.11, 79.53 (d, ^3^*J*_PCCC_ = 7.4 Hz, C5), 63.38 (d, ^1^*J*_PC_ = 169.5 Hz, C3), 63.13 (d, ^2^*J* = 6.6 Hz, *C*OP), 62.75 (d, ^2^*J* = 6.6 Hz, *C*OP), 62.66 (d, ^3^*J* = 3.4 Hz, *C*H_2_N), 60.59 (d, ^3^*J* = 7.5 Hz, *C*H_2_C4), 59.33 (*C*H_2_C5), 49.72 (C4), 16.19 (d, ^3^*J* = 5.7 Hz, *C*COP), 16.13 (d, ^3^*J* = 5.7 Hz, *C*COP). ^31^P NMR (243 MHz, CDCl_3_): δ = 22.05 ppm. ^31^P NMR (243 MHz, C_6_D_6_): δ = 22.94. Analysis Calculated for C_16_H_26_NO_6_P: C, 53.48; H, 7.29; N, 3.90. Found: C, 53.25; H, 7.16; N, 4.20.

*2-Benzyl-3-(diethoxyphosphoryl)isoxazolidine-4,5-dicarboxylic acid (***23***)***.** Compound **23** was prepared from nitrone **22** (2.00 mmol, 0.542 g) and maleic anhydride (2.20 mmol, 0.146 mL) and purified by column chromatography on a silica gel column with chloroform-methanol (10:1, *v*/*v*). Yield: 74% (0.573 g) as a yellowish oil. IR (film, cm^−1^): ν_max_= 3040, 2984, 2925, 1738, 1494 1206, 1045, 1020. ^1^H NMR (600 MHz, CDCl_3_): δ = 7.40–7.38 (m, 2H, *H_Ar_*), 7.32–7.28 (m, 2H, *H_Ar_*), 7.25–7.20 (m, 1H, *H_Ar_*), 6.50–6.10 (br s, 2H, 2 × OH), 4.76 (d, ^3^*J*_H5–H4_ = 8.3 Hz, 1H, *H*C5), 4.41 (d, ^2^*J*_Ha–Hb_=14.0 Hz, 1H, *H*_a_H_b_CPh), 4.23 (d, ^2^*J*_Ha–Hb_=14.0 Hz, 1H, H_a_*H*_b_CPh), 4.25–4.15 (m, 4H, 2 × C*H*_2_OP), 4.02 (ddd, ^3^*J*_H4–P_ = 16.3 Hz, ^3^*J*_H4–H5_ = 8.3 Hz,^3^*J*_H4–H3_ = 8,0 Hz, 1H, *H*C4), 3.89 (dd, ^3^*J*_H3–H4_ = 8.0 Hz, ^2^*J*_H3–P_ = 3.1 Hz, 1H, *H*C3), 1.33 (t, ^3^*J* = 7.1 Hz, 3H, C*H*_3_CH_2_OP), 1.31 (t, ^3^*J*= 7.1 Hz, 3H, C*H*_3_CH_2_OP). ^13^C NMR (151 MHz, CDCl_3_): δ = 172.48 (C=O), 171.97 (C*=*O), 137.11, 129.10, 128.28, 127.42, 77.78 (br s, C5), 64.29 (d, ^2^*J* = 6.6 Hz, *C*OP), 64.11 (d, ^1^*J*_PC_ = 170.8 Hz, C3), 64.00 (d, ^2^*J* = 6.4 Hz, *C*OP), 62.74 (br s, *C*H_2_N), 53.43 (C4), 16.42 (d, ^3^*J* = 5.6 Hz, *C*COP), 16.31 (d, ^3^*J* = 5.6 Hz, *C*COP). ^31^P NMR (243 MHz, CDCl_3_): δ = 19.85 ppm. Analysis Calculated for C_16_H_22_NO_8_P: C, 49.62; H, 5.73; N, 3.62. Found: C, 49.90; H, 5.52; N, 3.86.

### 3.3. Preparation of γ-Lactam ***18***

**Procedure A:** A solution of isoxazolidine **20** (0.045 g, 0.108 mmol) in methanol (1 mL) was kept under an atmospheric pressure of hydrogen over 20% Pd(OH)_2_–C (1.4 mg) at room temperature for 24 h. The suspension was filtered through a layer of Celite. The solution was concentrated, and the residue was chromatographed on silica gel column with chloroform-methanol (100:1, *v*/*v*) to give pure γ-lactam **18** (0.024 g, 0.081 mmol, 75%).

**Procedure B:** A solution of isoxazolidine **20** (0.208 g, 0.50 mmol) in methanol (5 mL) was kept in a pressure reactor under 15 bar pressure of hydrogen over 20% Pd(OH)_2_-C (6.5 mg) at room temperature for 5 h. The suspension was filtered through a layer of Celite. The solution was concentrated, and the residue was chromatographed on a silica gel column with chloroform-methanol (100:1, *v*/*v*) to give pure γ-lactam **18** (0.138 g, 94%).

Methyl 2-(diethoxyphosphoryl)-4-hydroxy-5-oxopyrrolidine-3-carboxylate (**18**)**.** White solid; m.p. = 120–124 °C. IR (KBr, cm^−1^): ν_max_= 3298, 3189, 3126, 2989, 2957, 2925, 2796, 1741, 1713, 1441, 1374, 1248, 1170, 1049, 1010, 862, 753. ^1^H NMR (600 MHz, CDCl_3_): δ = 7.15 (brs, 1H, N*H*), 4.54 (d, ^3^*J*_H3–OH_ = 7.6 Hz, 1H, OH), 4.46 (dd, ^3^*J*_H3–H4_ = 8.8 Hz, ^3^*J*_H3–OH_ = 7.6 Hz, 1H, *H*C4), 4.25–4.14 (m, 4H, 2 × C*H*_2_OP), 4.07 (dd, ^3^*J*_H2–H3_ = 8.8 Hz, ^2^*J*_H2–P_ = 3.2 Hz, 1H, *H*C2), 3.79 (s, 3H, C*H**_3_***O), 3.40 (dt, ^3^*J*_H3–P_ = 17.6 Hz, ^3^*J*_H4–H3_ = 8.8 Hz, ^3^*J*_H3–H2_ = 8.8 Hz, 1H, *H*C3), 1.35 (t, ^3^*J* = 7.0 Hz, 3H, C*H*_3_CH_2_OP), 1.32 (t, ^3^*J*= 7.0 Hz, 3H, C*H*_3_CH_2_OP). ^13^C NMR (151 MHz, CDCl_3_): δ = 174.94 (d, ^3^*J*_PCNC_ = 7.8 Hz, C5), 171.35 (d, ^3^*J*_PCCC_ = 2.3 Hz, *C*(O)CH_3_), 72.89 (d, ^3^*J*_PCCC_ = 7.8 Hz, C4), 63.77 (d, ^2^*J* = 7.1 Hz, *C*OP), 63.66 (d, ^2^*J* = 7.1 Hz, *C*OP), 52.82 (*C*H_3_O), 49.72 (d, ^2^*J*_CCP_ = 4.3 Hz, C3), 48.65 (d, ^1^*J*_CP_ = 167.1 Hz, *C*P), 16.41 (d, *J* = 6.0 Hz, *C*COP), 16.36 (d, *J* = 6.0 Hz, *C*COP). ^31^P NMR (243 MHz, CDCl_3_) δ = 20.63. Analysis Calculated for C_10_H_18_NO_7_P: C, 40.68; H, 6.15; N, 4.74. Found: C, 40.88; H, 6.23; N, 4.91.

### 3.4. Ammonolysis of ***18***

To a solution of γ-lactam **18** (0.030 g, 0.10 mmol) in methanol (0.5 mL), aqueous NH_3_, (25%, 0.4 mL) was added. The homogenous mixture was stirred at room temperature for 17 h. Solvents were removed in vacuo, and the residue was evaporated with anhydrous methanol (3 × 5 mL), chloroform (3 × 5 mL) and chromatographed on silica gel with chloroform-methanol (10:1, *v*/*v*) to give pure **24** (0.016 g, 60%).

2-(diethoxyphosphoryl)-4-hydroxy-5-oxopyrrolidine-3-carboxamide (**24**)**.** White solid; m.p. = 133–135 °C. IR (KBr, cm^−1^): ν_max_= 3300, 3199, 2986, 1711, 1679, 1231, 1045, 1020. ^1^H NMR (600 MHz, CD_3_OD): δ = 4.40 (d, ^3^*J*_H3-H4_ = 9.0 Hz, 1H, *H*C4), 4.25–4.20 (m, 4H, 2 × C*H*_2_OP), 4.08 (dd, ^3^*J*_H2–H3_ = 9.0 Hz, ^2^*J*_H2–P_ = 2.0 Hz, 1H, *H*C2), 3.18 (dt, ^3^*J*_H3–P_ = 18.0 Hz, ^3^*J*_H4–H3_ = 9.0 Hz, ^3^*J*_H3–H2_ = 9.0 Hz, 1H, *H*C3), 1.38 (t, ^3^*J* = 7.0 Hz, 6H, 2 × C*H*_3_CH_2_OP). ^13^C NMR (151 MHz, CD_3_OD): δ = 177.19 (d, ^3^*J*_PCNC_ = 7.9 Hz, C5), 174.65 (*C*(O)NH_2_), 74.11 (d, ^3^*J*_PCCC_ = 8.7 Hz, C4), 64.91 (d, ^2^*J* = 6.7 Hz, *C*OP), 64.82 (d, ^2^*J* = 6.7 Hz, *C*OP), 51.83 (d, ^2^*J*_CCP_ = 4.3 Hz, C3), 49.51 (d, ^1^*J*_CP_ = 167.4 Hz, *C*P), 16.74 (d, *J* = 5.6 Hz, *C*COP), 16.67 (d, *J* = 5.4 Hz, *C*COP). ^31^P NMR (243 MHz, CD_3_OD) δ = 21.25. Analysis Calculated for C_9_H_17_N_2_O_6_P: C, 38.58; H, 6.12; N, 10.00. Found: C, 38.29; H, 6.33; N, 9.92.

### 3.5. Mesylation of (Isoxazolidin-3-yl)Phosphonate **21**

To a solution of isoxazolidine **21** (0.188 g, 0.523 mmol) in methylene chloride (6 mL) triethylamine (1.569 mmol, 0.219 mL) and mesyl chloride (1.569 mmol, 0.122 mL) were added at 0°C. The reaction mixture was stirred at this temperature for 2 h. The residue was washed with water (3 × 3 mL) and dried over MgSO_4_. The solution was concentrated, and the residue was chromatographed on silica gel column with chloroform to give pure dimesylate **25** (0.250 g, 96%).

Diethyl 2-benzyl-4,5-(dimesyloxymethyl)-3-phosphonate (**25**)**.** Colourless oil. IR (film, cm^−1^): ν_max_= 3030, 2986, 2934, 1358, 1243, 1177, 1051, 1023, 964, 812, 754, 628. ^1^H NMR (600 MHz, CDCl_3_): δ = 7.39–7.33 (m, 4H, *H_Ar_*), 7.31–7.28 (m, 1H, *H_Ar_*), 4.59 (d, ^2^*J*_Ha–Hb_= 14.3 Hz, 1H, *H*_A_H_B_CPh), 4.46 (dd, ^2^*J*_Ha–Hb_ = 11.6 Hz, ^3^*J*_Ha–H4_ = 3.9 Hz, 1H, H_a_*H*_b_C-C4), 4.40 (dd, ^2^*J*_Ha–Hb_ = 11.6 Hz, ^3^*J*_Ha–H4_ = 6.8 Hz, 1H, *H*_a_H_b_C-C4), 4.33–4.21 (m, 7H, 2 × C*H*_2_OP, *H*C5, *H*_a_*H*_b_C-C5), 3.88 (d, ^2^*J*_HA–HB_=14.3 Hz, 1H, H_A_*H*_B_CPh), 3.27 (ddddd, ^3^*J*_H4–P_ = 17.3 Hz, ^3^*J*_H4–Ha_ = 6.8 Hz, ^3^*J*_H4–H5_ = 8.9 Hz, ^3^*J*_H4–H3_ = 6.6 Hz, ^3^*J*_H4–Hb_ = 3.9 Hz, 1H, *H*C4), 3.04 (s, 3H, C*H*_3_), 2.97 (dd, ^3^*J*_H3–H4_ = 6.6 Hz, ^2^*J*_H3–P_ = 3.1 Hz, 1H, *H*C3), 2.89 (s, 3H, C*H*_3_), 1.41 (t, ^3^*J* = 7.1 Hz, 3H, C*H*_3_CH_2_OP), 1.39 (t, ^3^*J*= 7.1 Hz, 3H, C*H*_3_CH_2_OP). ^13^C NMR (151 MHz, CDCl_3_): δ = 136.22, 129.30, 128.29, 127.65, 75.93 (d, ^3^*J*_PCCC_ = 7.6 Hz, C5), 66.55 (*C*H_2_-C4), (65.86 (d, ^3^*J* = 8.0 Hz, *C*H_2_-C4), 64.01 (d, ^2^*J* = 6.6 Hz, *C*OP), 63.40 (d, ^1^*J*_PC_ = 170.7 Hz, C3), 62.95 (d, ^2^*J* = 6.6 Hz, *C*OP), 62.36 (d, ^3^*J* = 3.2 Hz, *C*H_2_N), 46.63 (d, ^2^*J* = 2.0 Hz, C4), 37.65 (*C*H_3_S), 37.57 (*C*H_3_S), 16.64 (d, ^3^*J* = 5.5 Hz, *C*COP), 16.48 (d, ^3^*J* = 5.5 Hz, *C*COP). ^31^P NMR (243 MHz, CDCl_3_): δ = 19.73. Analysis Calculated for C_18_H_30_NO_10_PS_2_: C, 41.94; H, 5.87; N, 2.72. Found C, 42.05; H, 5.93; N, 3.01.

### 3.6. The Synthesis of (Pyrrolidin-2-yl)Phosphonate ***24*** from Dimesylate ***25***

**Procedure A:** A solution of dimesylate **25** (0.20 g, 0.39 mmol) in methanol (2.5 mL) was kept under atmospheric pressure of hydrogen over 20% Pd(OH)_2_-C (3.9 mg) at room temperature for 22 h. The reaction progress was controlled by TLC. The suspension was filtered through a layer of Celite. The solution was concentrated to give ammonium mesylate (**26**), which was then dissolved in chloroform (4 mL) and anhydrous potassium carbonate (0.108 g, 0.78 mmol) was added. The reaction mixture was stirred at room temperature for 3 h. Then anhydrous MgSO_4_ was added, and the suspension was filtered through a layer of Celite. The solution was concentrated, and the residue was chromatographed on silica gel column with chloroform-methanol (100:1, *v*/*v*) to give pure **27** (0.049 g, 38%).

**Procedure B:** A solution of dimesylate **25** (0.236 g, 0.46 mmol) in methanol (5 mL) was kept in a pressure reactor under 15 bar pressure of hydrogen over 20% Pd(OH)_2_-C (10 mg) at room temperature for 6 h. The suspension was filtered through a layer of Celite. The solution was concentrated to give ammonium mesylate (**26**), which was then dissolved in chloroform (10 mL) and anhydrous potassium carbonate (0.127 g, 0.92 mmol) was added. The reaction mixture was stirred at room temperature for 3 h. Then anhydrous MgSO_4_ was added, and the suspension was filtered through a layer of Celite. The solution was concentrated, and the residue was chromatographed on silica gel column with chloroform-methanol (100:1, *v*/*v*) to give pure **27** (0.106 g, 70%).

Diethyl 4-hydroxy-3-mesyloxymethyl(pyrrolidin-2-yl)-phosphonate (**27**)**.** Colorless oil. IR (film, cm^−1^): ν_max_= 3386, 2986, 2875, 1644, 1352, 1222, 1174, 1025, 970, 818. ^1^H NMR (600 MHz, CDCl_3_): δ = 4.31 (dd, *J* = 10.2 Hz, *J* = 5.6 Hz, 1H, *H*CH-C3), 4.25–4.15 (m, 6H, *H*C4, HC*H*-C3, 2 × C*H_2_*OP), 3.26 (dd, ^2^*J*_H2–P_ = 7.9 Hz, ^3^*J*_H2–H3_ = 5.8 Hz, 1H, *H*C2), 3.17 (dd, ^2^*J*_Ha–Hb_ = 11.5 Hz, ^3^*J*_H5–H_ = 5.3 Hz, 1H, *H*HC5), 3.10 (dd, ^2^*J*_Ha–Hb_ = 11.4 Hz, ^3^*J*_H5–H_ = 3.0 Hz, 1H, H*H*C5), 3.06 (s, 3H, C*H_3_*S), 2.65 (ddddd, ^3^*J*_H3–P_ = 18.5 Hz, ^3^*J*_H3–H2_ = 5.8 Hz, ^3^*J*_H3–H4_ = 5.8 Hz, ^3^*J*_H3–H_ = 5.6 Hz, ^3^*J*_H3–H_ = 2.7 Hz, 1H, *H*C3), 1.36 (t, ^3^*J* = 7.1 Hz, 3H, C*H*_3_CH_2_OP), 1.35 (t, ^3^*J* = 7.1 Hz, 3H, C*H*_3_CH_2_OP). ^1^H NMR (600 MHz, CD_3_OD): δ = 4.45 (dd, *J* = 10.3 Hz, *J* = 4.3 Hz, 1H, C*H_2_*-C3), 4.35 (dd, *J* = 10.3 Hz, *J* = 5.2 Hz, 1H, C*H_2_*-C3), 4.27–4.20 (m, 4H, 2 × C*H_2_*OP), 4.18 (ddd, *J* = 5.9 Hz, *J* = 5.8 Hz, *J* = 5.7 Hz, 1H, *H*C4), 3.30 (dd, ^2^*J*_H2–P_ = 8.9 Hz, ^3^*J*_H2–H3_ = 8.8 Hz, 1H, *H*C2), 3.15 (s, 3H, C*H_3_*S), 3.06 (dd, ^2^*J*_Ha–Hb_ = 11.5 Hz, ^3^*J*_H5–H_ = 8.9 Hz, 1H, *H*HC5), 2.86 (ddd, ^2^*J*_Ha–Hb_ = 11.5 Hz, ^3^*J*_H5–H_ = 5.7 Hz, ^3^*J*_H5–CN–H_ = 1.1 Hz, 1H, H*H*C5), 2.47 (ddddd, ^3^*J*_H3–P_ = 18.0 Hz, ^3^*J*_H3–H2_ = 8.8 Hz, ^3^*J*_H3–H4_ = 5.8 Hz, ^3^*J*_H3–H_ = 5.2 Hz, ^3^*J*_H3–H_ = 4.3 Hz, 1H, *H*C3), 1.39 (t, ^3^*J* = 7.1 Hz, 3H, C*H_3_*CH_2_OP), 1.38 (t, ^3^*J* = 7.1 Hz, 3H, C*H_3_*CH_2_OP). ^13^C NMR (151 MHz, CD_3_OD): δ = 74.65 (d, ^3^*J*_PCCC_ = 7.9 Hz, C4), 69.44 (d, ^3^*J*_PCCC_ = 2.6 Hz, *C*OMs), 64.48 (d, *J* = 7.1 Hz, *C*OP), 64.21 (d, *J* = 7.2 Hz, *C*OP), 55.35 (d, ^1^*J*_PC_ = 167.5 Hz, C2), 55.09 (d, ^3^*J*_PCNC_ = 8.9 Hz, C5), 50.19 (C3), 37.13 (***C***H_3_S), 16.80 (d, *J* = 5.6 Hz, POC*C*), 16.80 (d, *J* = 5.7 Hz, POC*C*). ^31^P NMR (243 MHz, CDCl_3_): δ = 26.33. Analysis Calculated for C_10_H_22_NO_7_PS: C, 36.25; H, 6.69; N, 4.23. Found: C, 36.46; H, 6.96; N, 4.20.

### 3.7. Synthesis of (Pyrrolidin-2-yl)Phosphonate ***28***

(Pyrrolidin-2-yl)-phosphonate **27** (0.040 g, 0.12 mmol) with morpholine (1.5 mL) was kept at room temperature for 39 h. After that chloroform (15 mL) was added. The solution was washed with water (2 × 10 mL), dried over MgSO_4_ and concentrated. The residue was chromatographed on silica gel column with chloroform-methanol (100:1, 50:1, *v*/*v*) to give pure **28** (0.014, 33%).

Diethyl (4-hydroxy-3-(piperidin-1-ylmeylyl)pyrrolidin-2-yl)phosphonate (**28**)**.** Colourless oil. IR (film, cm^−1^): ν_max_= 3442, 2960, 2930, 2859, 2815, 1646, 1446, 1222, 1049, 1027. ^1^H NMR (600 MHz, CDCl_3_): δ = 4.30–4.15 (m, 4H, 2 × C*H_2_*OP), 4.08–4.05 (br m, 1H, *H*C4), 3.75–3.70 (br m, 4H), 3.37 (dd, ^2^*J*_H2–P_ = 5.4 Hz, ^3^*J*_H2–H3_ = 5.4 Hz, 1H, *H*C2), 3.27 (dd, ^2^*J*_Ha–Hb_ = 10.9 Hz, ^3^*J*_H5–H_ = 5.6 Hz, 1H, *H*HC5), 3.03 (dd, ^2^*J*_Ha–Hb_ = 10.9 Hz, ^3^*J*_H5–H_ = 4.5 Hz, 1H, H*H*C5), 2.60–2.45 (m, 7H, *H*C3, 3 × C*H*_2_-C3), 1.38 (t, ^3^*J* = 7.1 Hz, 3H, C*H_3_*CH_2_OP), 1.36 (t, ^3^*J* = 7.1 Hz, 3H, C*H_3_*CH_2_OP). ^13^C NMR (151 MHz, CDCl_3_): δ = 77.16 (d, ^3^*J*_PCCC_ = 5.4 Hz, C4), 66.69 (*C*H_2_-O-*C*H_2_), 61.11 (d, ^3^*J*_PCCC_ = 8.8 Hz, *C*H_2_N), 63.12 (d, *J* = 7.4 Hz, *C*OP), 62.37 (d, *J* = 7.5 Hz, *C*OP), 55.64 (d, ^1^*J*_PC_ = 166.1 Hz, C2), 53.75 (2 × *C*H_2_N), 53.60 (d, ^3^*J*_PCNC_ = 6.6 Hz, C5), 45.40 (C3), 16.62 (d, *J* = 5.5 Hz, POC*C*), 16.54 (d, *J* = 5.9 Hz, POC*C*). ^31^P NMR (243 MHz, CDCl_3_): δ = 28.04. Analysis Calculated for C_13_H_27_N_2_O_5_P: C, 48.44; H, 8.44; N, 8.69. Found: C, 48.27; H, 8.48; N, 8.93.

### 3.8. Synthesis of azide ***29***

To a solution of mesylate **27** (0.100 g, 0.30 mmol) in methanol (2 mL) sodium azide was added (0.060 g, 0.90 mmol). The reaction mixture was stirred at 60°C for 96 h. The solvent was removed and the reside was chromatographed on silica gel column with chloroform-methanol (100:1, 50:1, *v*/*v*) to give pure azide **29** (0.036 g, 43%).

Diethyl (3-(azidomethyl)-4-hydroxypyrrolidin-2-yl)phosphonate (**29**)**.** Colourless oil. IR (film, cm^−1^): ν_max_= 3355, 2985, 2933, 2872, 2103, 1648, 1445, 1354, 1225, 1175, 1026, 970. ^1^H NMR (600 MHz, CDCl_3_): δ = 4.26–4.13 (m, 4H, 2 × C*H_2_*OP), 4.11 (ddd, ^3^*J*_H4–H5_ = 5.2 Hz, ^3^*J*_H4–H5_ = 2.5 Hz, ^3^*J*_H4–H3_ = 2.5 Hz, 1H, *H*C4), 3.45 (ddd, ^2^*J*_H–H_ = 12.2 Hz, ^2^*J*_H–H3_ = 6.7 Hz, ^4^*J*_H–P_ = 0.9 Hz, 1H, *H*CHN_3_), 3.40 (dd, ^2^*J*_H–H_ = 12.2 Hz, ^2^*J*_H–H3_ = 6.7 Hz, 1H, HC*H*N_3_), 3.23 (dd, ^2^*J*_H2–P_ = 7.2 Hz, ^3^*J*_H2–H3_ = 4.8 Hz, 1H, *H*C2), 3.17 (dd, ^2^*J*_Ha–Hb_ = 11.3 Hz, ^3^*J*_H5–H4_ = 5.2 Hz, 1H, *H*HC5), 3.07 (dd, ^2^*J*_Ha–Hb_ = 11.3 Hz, ^3^*J*_H5–H4_ = 2.5 Hz, 1H, H*H*C5), 2.50 (ddddd, ^3^*J*_H3–P_ = 14.8 Hz, ^3^*J*_H3–H_ = 6.7 Hz, ^3^*J*_H3–H_ = 6.7 Hz, ^3^*J*_H3–H2_ = 4.8 Hz, ^3^*J*_H3–H4_ = 2.5 Hz, 1H, *H*C3), 1.36 (t, ^3^*J* = 7.1 Hz, 6H, 2 × C*H_3_*CH_2_OP). ^13^C NMR (151 MHz, CDCl_3_): δ = 75.36 (d, ^3^*J*_PCCC_ = 5.5 Hz, C4), 63.36 (d, *J* = 6.9 Hz, *C*OP), 62.74 (d, *J* = 7.0 Hz, *C*OP), 55.94 (d, ^1^*J*_PC_ = 164.1 Hz, C2), 54.87 (d, ^3^*J*_PCNC_ = 8.6 Hz, C5), 52.48 (d, ^3^*J*_PCCC_ = 10.5 Hz, CH_2_N_3_), 49.35 (C3), 16.57 (d, *J* = 5.5 Hz, POC*C*), 16.53 (d, *J* = 5.5 Hz, POC*C*). ^31^P NMR (243 MHz, CDCl_3_): δ = 27.42. Analysis Calculated for C_9_H_19_N_4_O_4_P: C, 38.85; H, 6.88; N, 20.14. Found: C, 39.03; H, 6.94; N, 20.11.

### 3.9. Synthesis of (Pyrrolidin-2-yl)Phosphonate ***30***

A solution of azide **29** (0.036 g, 0.13 mmol) Boc_2_O (0.125 g, 0.572 mmol) in ethanol (0.5 mL) was kept under atmospheric pressure of hydrogen over 20% Pd(OH)_2_-C (1.7 mg) at room temperature for 35 h. The suspension was filtered through a layer of Celite. The solution was concentrated, and the residue was chromatographed on silica gel column with chloroform-methanol (100:1, 50:1, *v*/*v*) to give pure **27** (0.050 g, 69%).

*tert*-butyl 3-{[(*tert*-butoxycarbonyl)amino]methyl}-2-(diethoxyphosphoryl)-4-hydroxypyrrolidine-1-carboxylate (**30**)**.** Colorless oil. IR (film, cm^−1^): ν_max_= 3312, 2980, 2933, 1743, 1703, 1519, 1279, 1254, 1165, 1028. ^1^H NMR (600 MHz, CDCl_3_): δ = 5.40–5.00 (br s, 1H, OH), 4.80–4.72 (br m, 1H, *H*C4), 4.25–4.15 (br m, 5H, 2 × C*H_2_*OP and *H*CHN), 4.10–4.00 (br m, 1H, HC*H*N), 3.48–3.40 (br m, 1H, *H*CH5), 3.28–3.20 (br m, 2H, *H*C2 and HC*H*C5), 2.85–2.70 (m, 1H, *H*C3), 1.50 (s, 9H, (C*H_3_*)_3_C), 1.48 (s, 9H, (C*H_3_*)_3_C), 1.46 (br s, 9H, (C*H_3_*)_3_C), 1.35 (t, *J* = 7.0 Hz, 6H, 2 × C*H_3_*CH_2_OP). ^13^C NMR (151 MHz, CDCl_3_): δ = 165.12 (C=O), 153.75 (C=O), 152.98 (C=O), 82.86 (*C*(CH_3_)_3_), 80.78 (*C*(CH_3_)_3_), 79.37 (*C*(CH_3_)_3_), 76.01 (very broad s, C4, 60%) and 75.13 (very broad s, C4, 40%), 62.83 (d, *J* = 7.2 Hz, *C*H_2_OP), 62.53 (br d, *J* ~ 7 Hz, *C*H_2_OP), 55.76 (very broad d, *J* ~ 167 Hz, C2, 40%) and 54.75 (very broad d, *J* ~ 164 Hz, C2, 60%), 50.47 (very broad s, C5, 60%) and 50.11 (very broad s, C5, 40%), 47.00 (very broad s, *C*H_2_N, 40%) and 45.45 (very broad s, *C*H_2_N, 60%), 42.46 (C3, 60%) and 41.93 (C3, 40%), 28.37, 28.26, 27.71, 16.48 (d, *J* = 5.7 Hz, POC*C*), 16.43 (very broad s, POC*C*). ^31^P NMR (243 MHz, CDCl_3_): δ = 24.06. Analysis Calculated for C_24_H_45_N_2_O_10_P: C, 52.16; H, 8.21; N, 5.07. Found: C, 52.32; H, 8.14; N, 5.02.

## 4. Conclusions

The 1,3-dipolar cycloadditions of *N*-benzyl-*C*-(diethoxyphosphoryl)nitrone **22** with dimethyl maleate and *cis*-1,4-dihydroxybut-2-ene, as well as maleic anhydride proceeded diastereospecifically to give cycloadducts **20**, **21** and **23**, respectively. Isoxazolidine **20** was smoothly hydrogenated to substituted (5-oxopyrrolidin-2-yl)phosphonate **18** and subsequently transformed into derivative **24** by exchanging of COOMe at C3 into amido function. For transformation of isoxazolidine **21** into functionalized derivative of (pyrrolidin-2-yl)phosphonate **28**, reaction sequence consisted of a standard mesylation of both hydroxy groups, a hydrogenolytic cleavage of the N–O bond, removal of benzyl group followed by spontaneous formation of the pyrrolidine ring by intramolecular S_N_2 reaction and finally exchanging the other mesyloxy group to amino function. Since 3-methoxycarbonyl-(5-oxopyrrolidin-2-yl)phosphonate **18** and 3-mesyloxymethyl(pyrrolidin-2-yl)phosphonate **27** contain reactive groups, i.e., COOMe at C3 in **18** and MsO at C3 in **27**, studies on their further functionalization based on their reactions with other nucleophiles are underway in our laboratory. The presented methodology could also be adopted for the synthesis of other stereoisomeric isoxazolidines via application of respective *trans*-alkenes in 1,3-dipolar cycloaddition to C-phosphorylated nitron **22**. Moreover, the syntheses elaborated herein pave the way for new enantiomerically pure functionalized phosphonate analogues of prolines, substituted glutamic acid by application of the *N*-chiral *C*-phosphorylated nitrones.

## Data Availability

Data is contained within the article.

## Supplementary Materials

Figure S1: ^1^H NMR Spectrum for **20** in CDCl_3_, Figure S2: ^13^C NMR Spectrum for **20** in CDCl_3_, Figure S3: ^31^P NMR Spectrum for **20** in CDCl_3_, Figure S4: ^1^H NMR Spectrum for **21** in CDCl_3_, Figure S5: ^1^H NMR Spectrum for **21** in C_6_D_6_, Figure S6: ^13^C NMR Spectrum for **21** in C_6_D_6_, Figure S7: ^31^P NMR Spectrum for **21** in CDCl_3_, Figure S8: ^31^P NMR Spectrum for **21** in C_6_D_6_, Figure S9: ^1^H NMR Spectrum for **23** in CDCl_3_, Figure S10: ^13^C NMR Spectrum for **23** in CDCl_3_, Figure S11: ^31^P NMR Spectrum for **23** in CDCl_3_, Figure S12: ^1^H NMR Spectrum for **18** in CDCl_3_, Figure S13: ^13^C NMR Spectrum for **18** in CDCl_3_, Figure S14: ^31^P NMR Spectrum for **18** in CDCl_3_, Figure S15: ^1^H NMR Spectrum for **24** in CD_3_OD, Figure S16: ^13^C NMR Spectrum for **24** in CD_3_OD, Figure S17: ^31^P NMR Spectrum for **24** in CD_3_OD, Figure S18: ^1^H NMR Spectrum for **25** in CDCl_3_, Figure S19: ^13^C NMR Spectrum for **25** in CDCl_3_, Figure S20: ^31^P NMR Spectrum for **25** in CDCl_3_, Figure S21: ^1^H NMR Spectrum for **27** in CDCl_3_, Figure S22: ^1^H NMR Spectrum for **27** in CD_3_OD, Figure S23: ^13^C NMR Spectrum for **27** in CD_3_OD, Figure S24: ^31^P NMR Spectrum for **27** in CDCl_3_, Figure S25: ^1^H NMR Spectrum for **28** in CDCl_3_, Figure S26: ^13^C NMR Spectrum for **28** in CDCl_3_, Figure S27: ^31^P NMR Spectrum for **28** in CDCl_3_, Figure S28: ^1^H NMR Spectrum for **29** in CDCl_3_, Figure S29: ^13^C NMR Spectrum for **29** in CDCl_3_, Figure S30: ^31^P NMR Spectrum for **29** in CDCl_3_, Figure S31: ^1^H NMR Spectrum for **30** in CDCl_3_, Figure S32: ^1^H NMR Spectrum for **30** in CDCl_3_, Figure S33: ^31^P NMR Spectrum for **30** in CDCl_3_.

## Sample Availability

Samples are not available.
