# Supplementary material for: Synthesis of Functionalized Diethyl(pyrrolidin-2-yl)phosphonate and Diethyl(5-oxopyrrolidin-2-yl)phosphonate
Source: Molecules. 2021 May 25;26(11):3160. doi: 10.3390/molecules26113160 (PMC8197975; doi:10.3390/molecules26113160)
Supplement: Supplementary file 1 [file molecules-26-03160-s001.zip › molecules-1213217-supplementary.pdf]

Supplementary materials

Synthesis of Functionalized Diethyl (pyrrolidin-2-yl)Phosphonate and Diethyl (5-oxopyrrolidin-2-yl)Phosphonate

Iwona E. Głowacka *, Anna Hartwich, Iwona Rozpara and Dorota G. Piotrowska *

Bioorganic Chemistry Laboratory, Faculty of Pharmacy, Medical University of Lodz, 90-151 Lodz, Muszynskiego 1, Poland; anna.zdzienicka@umed.lodz.pl (A.H.); iwona.rozpara@umed.lodz.pl (I.R.)

* Correspondence: iwona.glowacka@umed.lodz.pl (I.E.G.); dorota.piotrowska@umed.lodz.pl (D.G.P.)

Contents

NMR spectra for compounds **20**, **21**, **23**, **18**, **24**, **25** and **27** – **30**

Figure S1: ^{1}H NMR Spectrum for **20** in $CDCl_3$

Figure S2: ^{13}C NMR Spectrum for **20** in $CDCl_3$

Figure S3: ^{31}P NMR Spectrum for **20** in $CDCl_3$

Figure S4: ^{1}H NMR Spectrum for **21** in $CDCl_3$

Figure S5: ^{1}H NMR Spectrum for **21** in C_6D_6

Figure S6: ^{13}C NMR Spectrum for **21** in C_6D_6

Figure S7: ^{31}P NMR Spectrum for **21** in $CDCl_3$

Figure S8: ^{31}P NMR Spectrum for **21** in C_6D_6

Figure S9: ^{1}H NMR Spectrum for **23** in $CDCl_3$

Figure S10: ^{13}C NMR Spectrum for **23** in $CDCl_3$

Figure S11: ^{31}P NMR Spectrum for **23** in $CDCl_3$

Figure S12: ^{1}H NMR Spectrum for **18** in $CDCl_3$

Figure S13: ^{13}C NMR Spectrum for **18** in $CDCl_3$

Figure S14: ^{31}P NMR Spectrum for **18** in $CDCl_3$

Figure S15: ^{1}H NMR Spectrum for **24** in CD_3OD

Figure S16: ^{13}C NMR Spectrum for **24** in CD_3OD

Figure S17: ^{31}P NMR Spectrum for **24** in CD_3OD

Figure S18: ^{1}H NMR Spectrum for **25** in $CDCl_3$

Figure S19: ^{13}C NMR Spectrum for **25** in $CDCl_3$

Figure S20: ^{31}P NMR Spectrum for **25** in $CDCl_3$

Figure S21: ^{1}H NMR Spectrum for **27** in $CDCl_3$

Figure S22: ^{1}H NMR Spectrum for **27** in CD_3OD

Figure S23: ^{13}C NMR Spectrum for **27** in CD_3OD

Figure S24: ^{31}P NMR Spectrum for **27** in $CDCl_3$

Figure S25: ^{1}H NMR Spectrum for **28** in $CDCl_3$

Figure S26: ^{13}C NMR Spectrum for **28** in $CDCl_3$

Figure S27: ^{31}P NMR Spectrum for **28** in $CDCl_3$

Figure S28: ^{1}H NMR Spectrum for **29** in $CDCl_3$

Figure S29: ^{13}C NMR Spectrum for **29** in $CDCl_3$

Figure S30: ^{31}P NMR Spectrum for **29** in $CDCl_3$

Figure S31: ^{1}H NMR Spectrum for **30** in $CDCl_3$

Figure S32: ^{13}C NMR Spectrum for **30** in $CDCl_3$

Figure S33: ^{31}P NMR Spectrum for **30** in $CDCl_3$

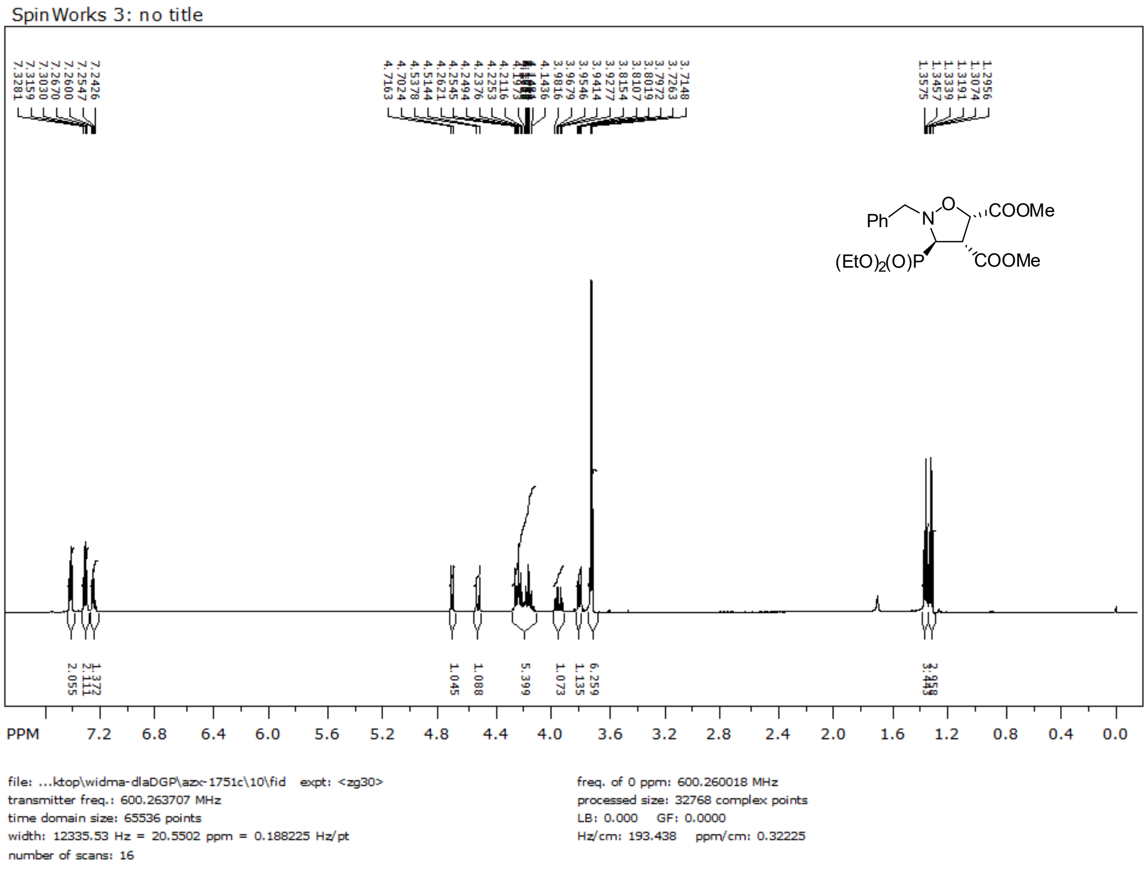

Figure S1: ^{1}H NMR Spectrum for **20** in $CDCl_3$

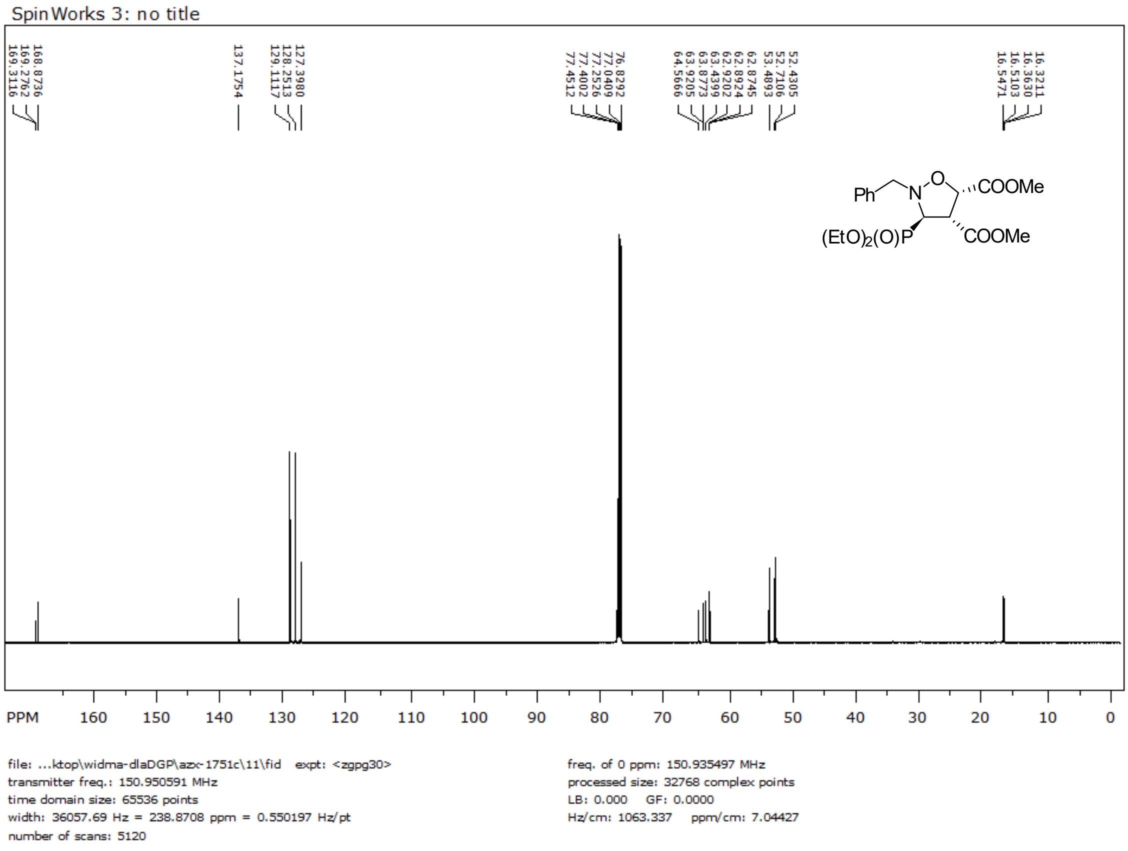

Figure S2: ^{13}C NMR Spectrum for **20** in $CDCl_3$

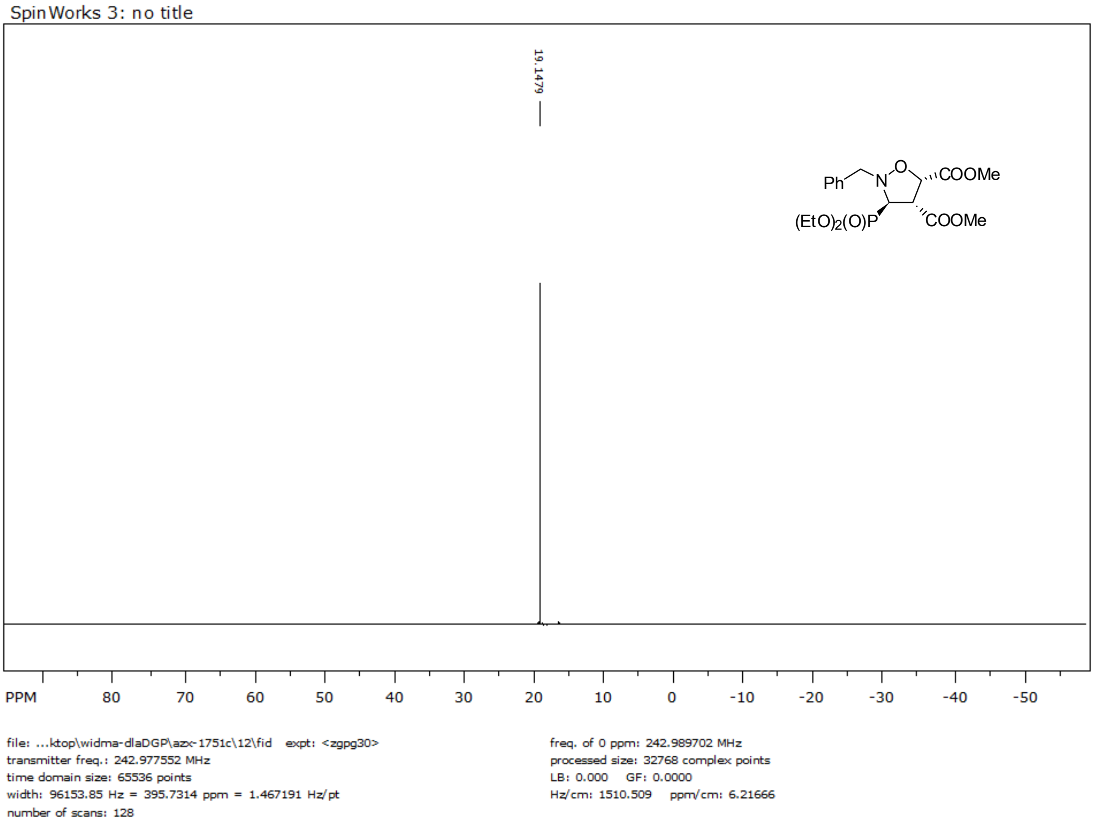

Figure S3: ^{31}P NMR Spectrum for **20** in $CDCl_3$

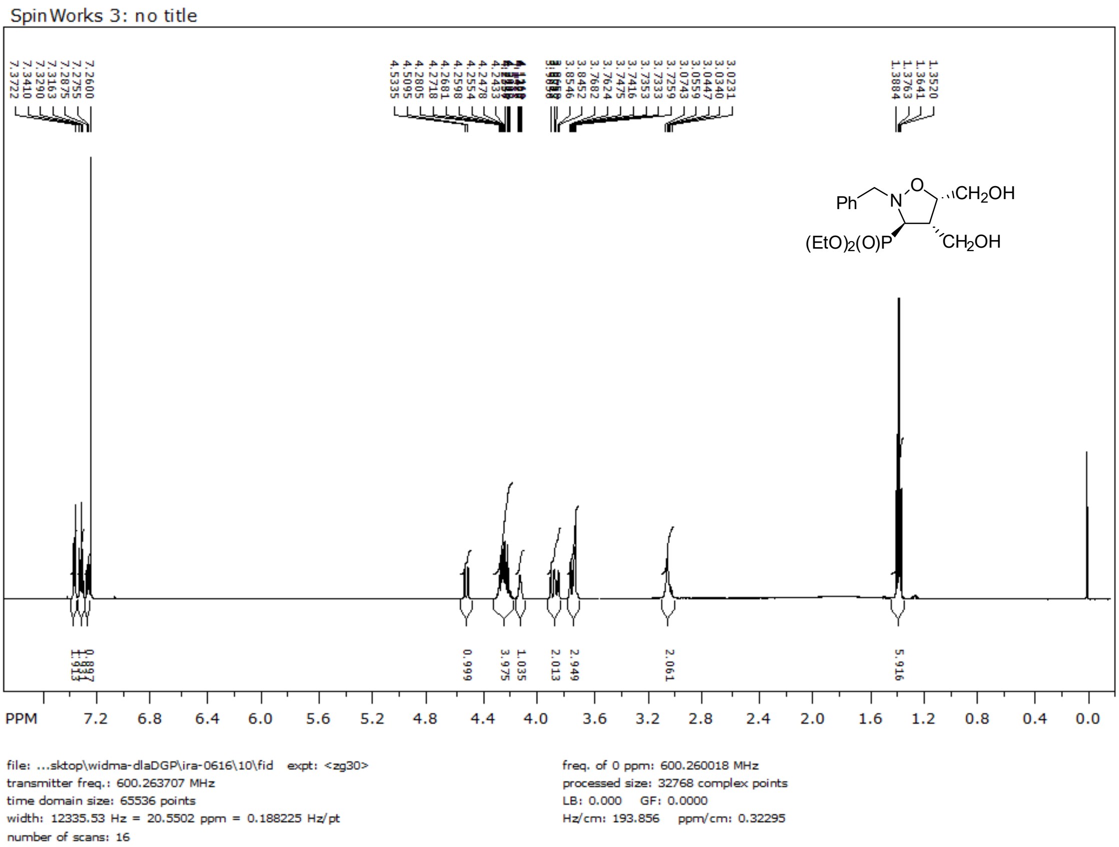

Figure S4: ^{1}H NMR Spectrum for **21** in $CDCl_3$

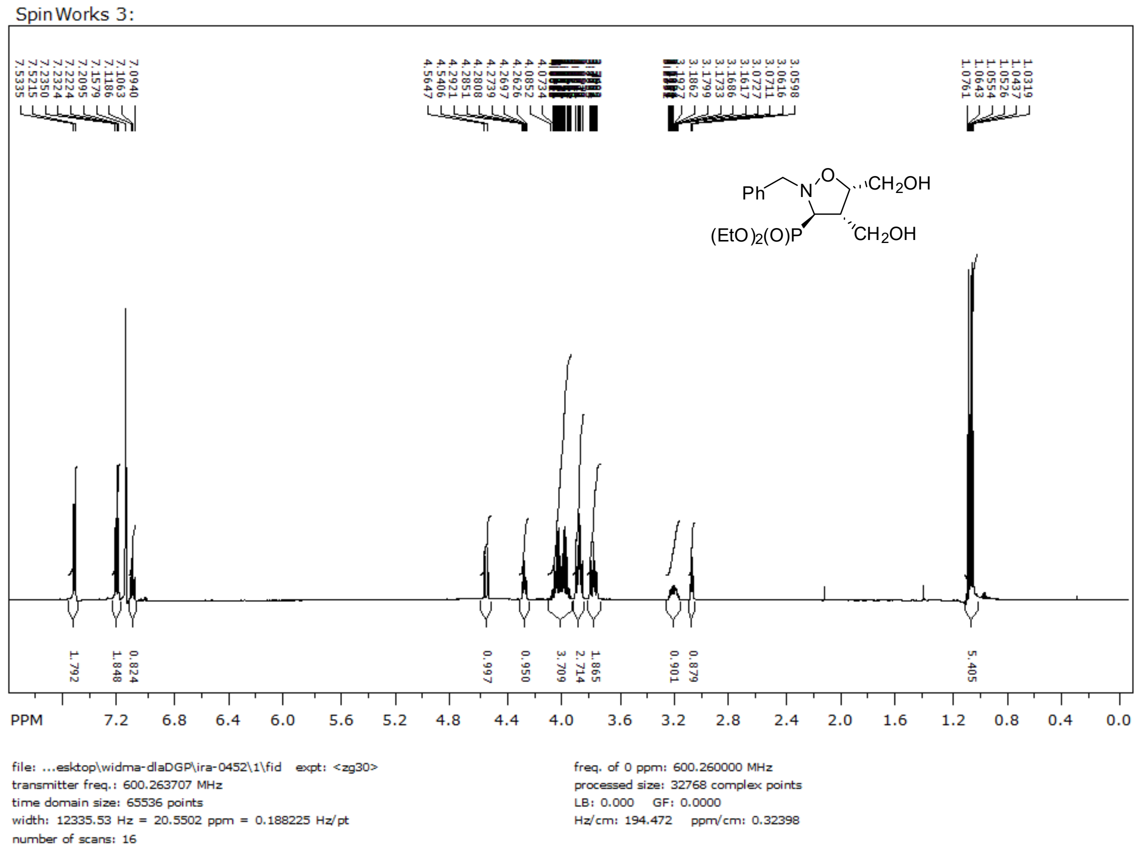

Figure S5: ^{1}H NMR Spectrum for **21** in C_6D_6

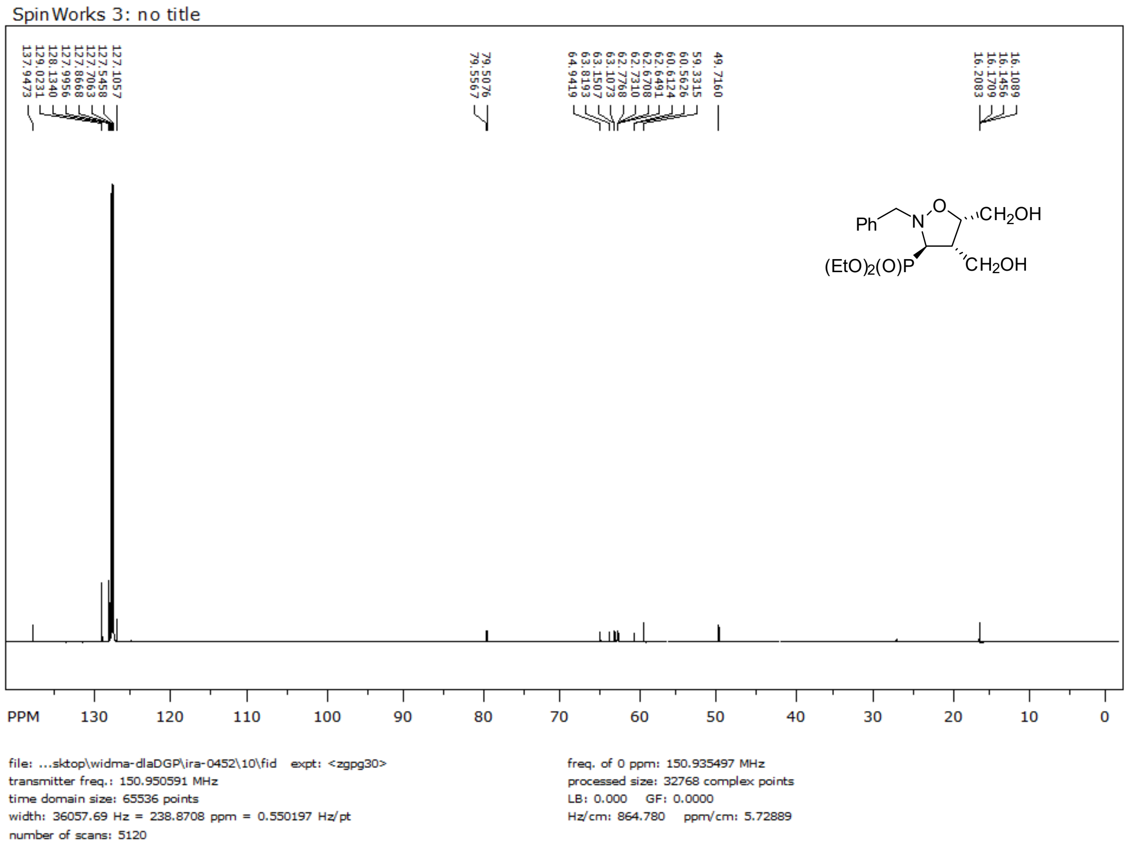

Figure S6: ^{13}C NMR Spectrum for **21** in C_6D_6

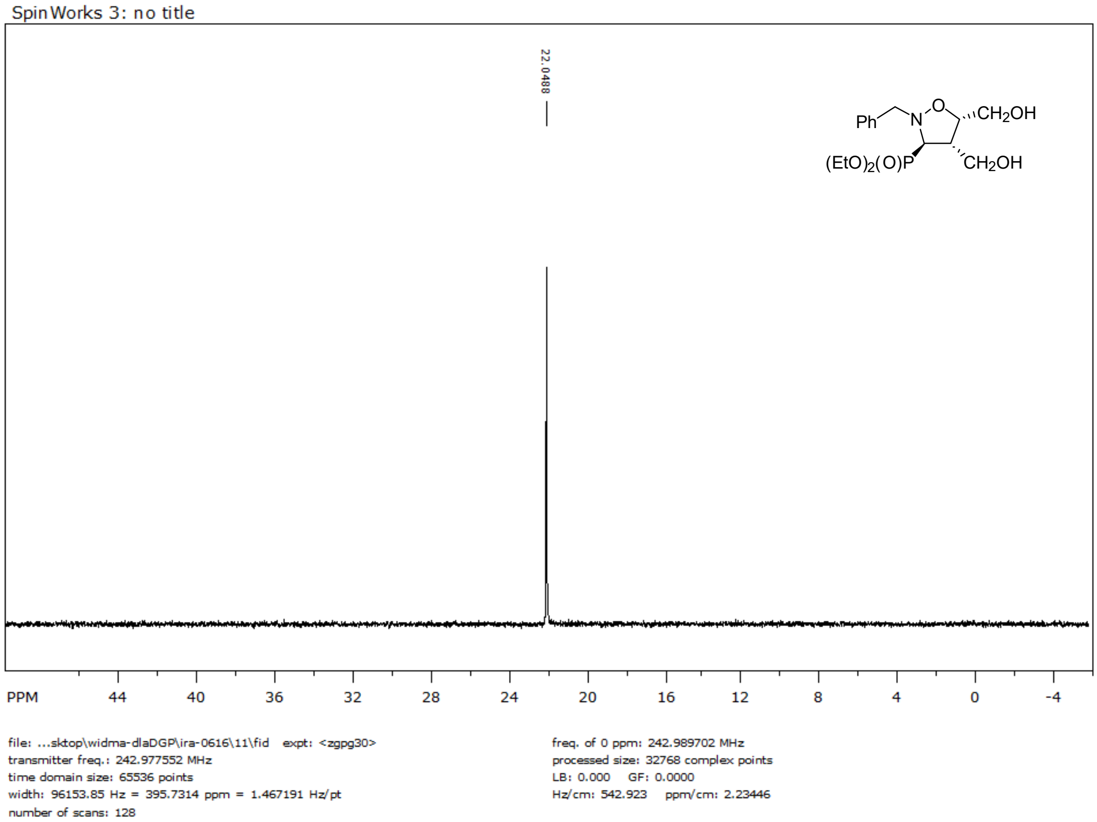

Figure S7: ^{31}P NMR Spectrum for **21** in $CDCl_3$

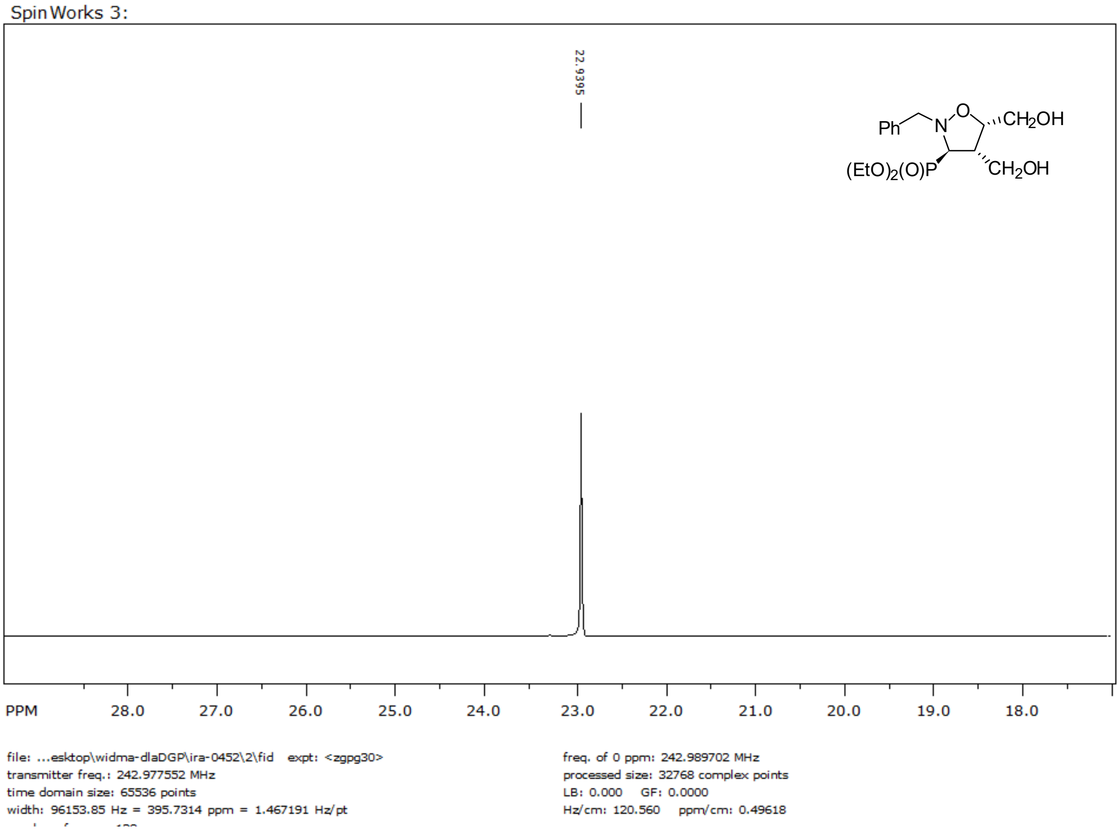

Figure S8: ^{31}P NMR Spectrum for **21** in C_6D_6

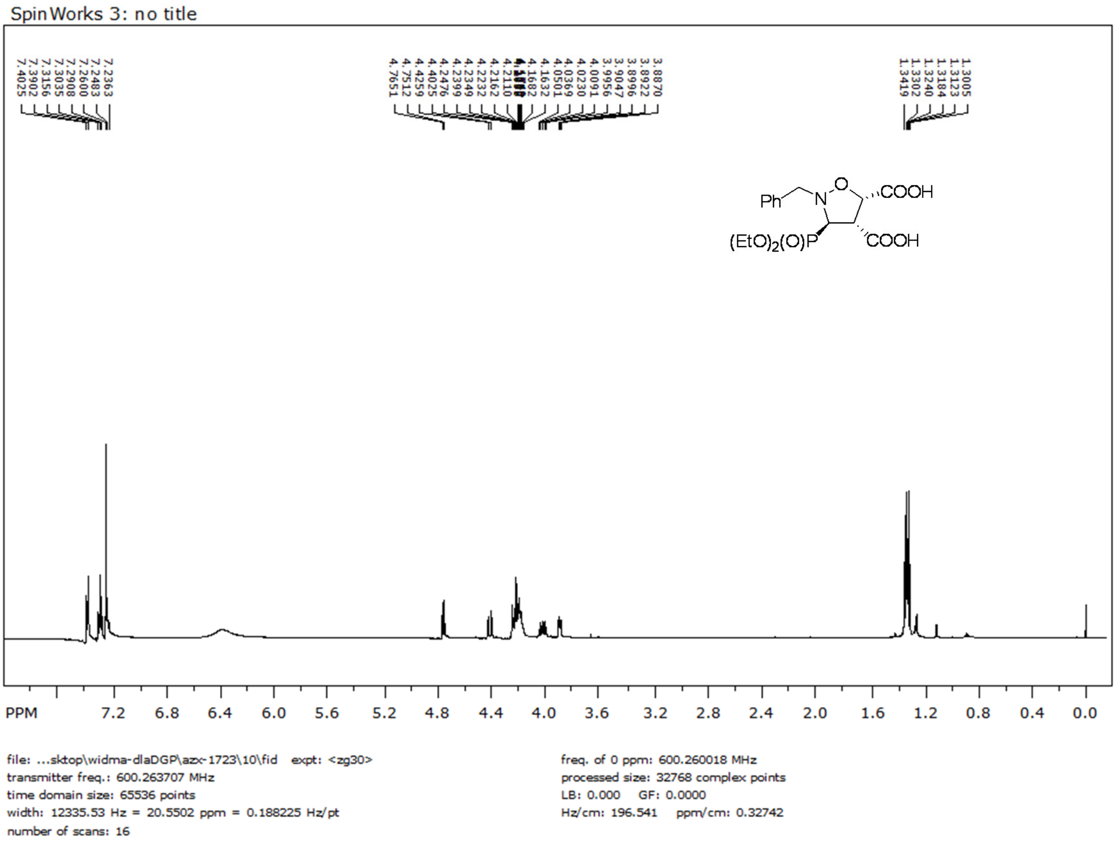

Figure S9: ¹H NMR Spectrum for **23** in $CDCl_3$

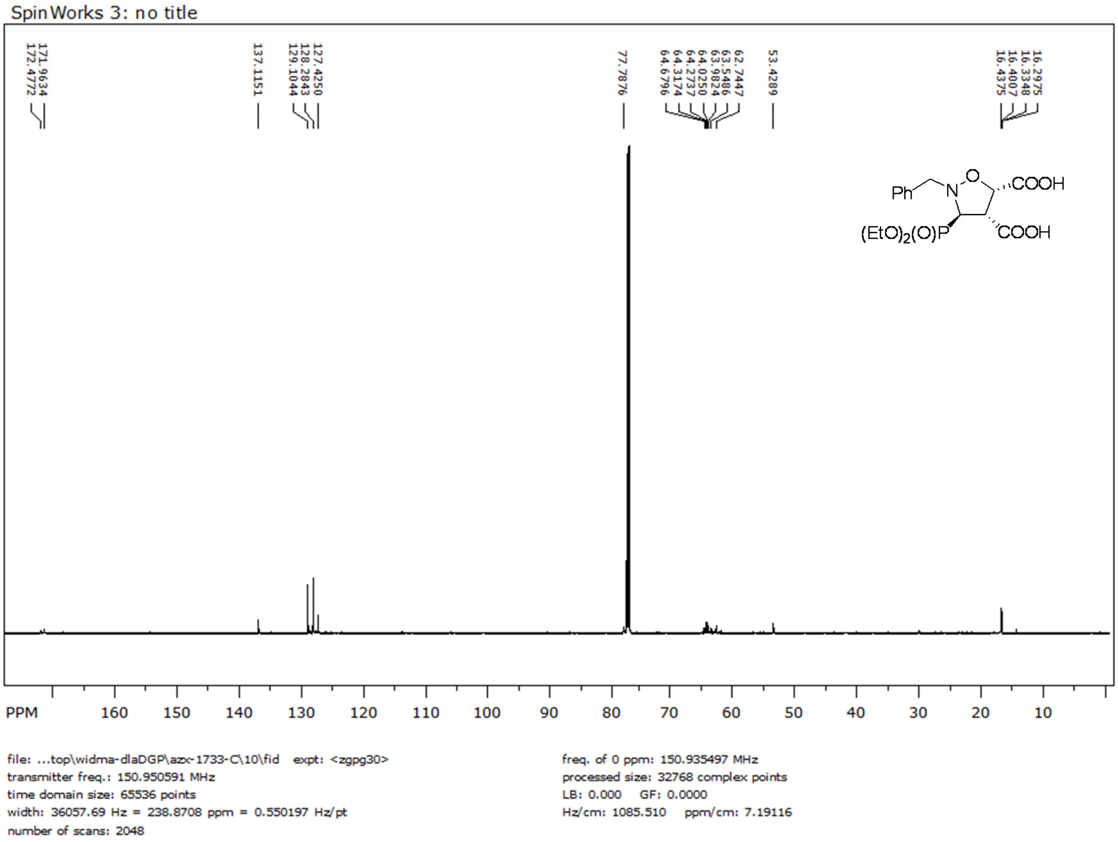

Figure S10: ¹³C NMR Spectrum for **23** in $CDCl_3$

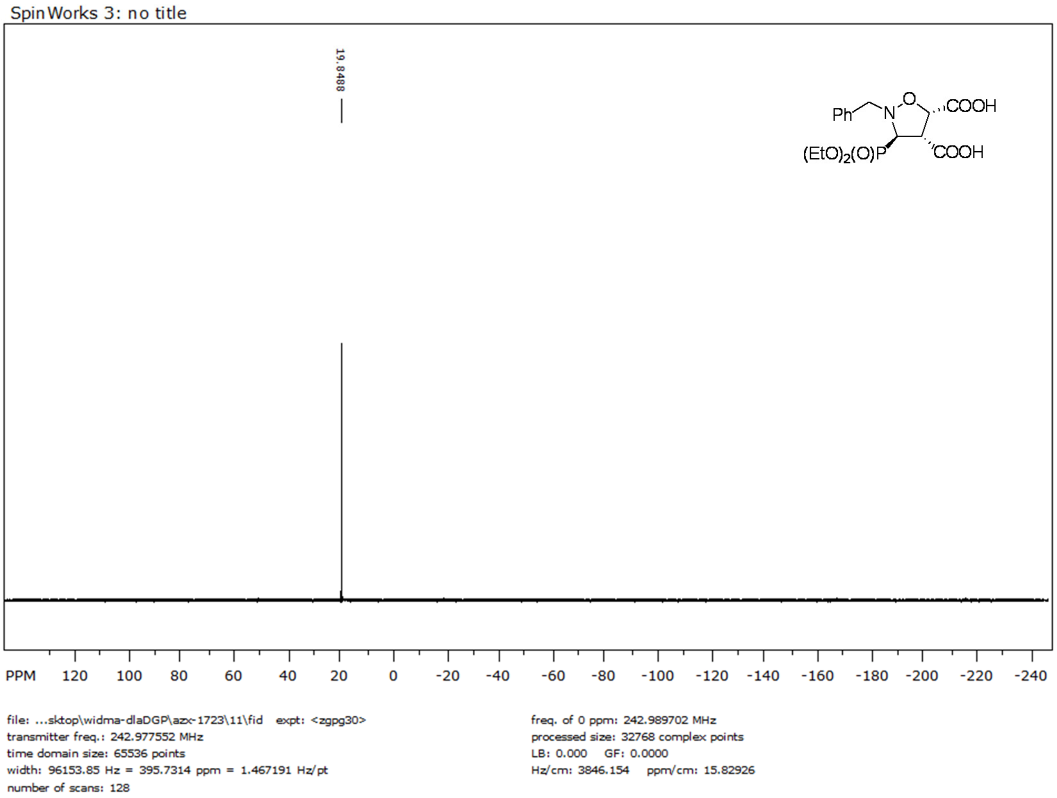

Figure S11: ^{31}P NMR Spectrum for **23** in $CDCl_3$

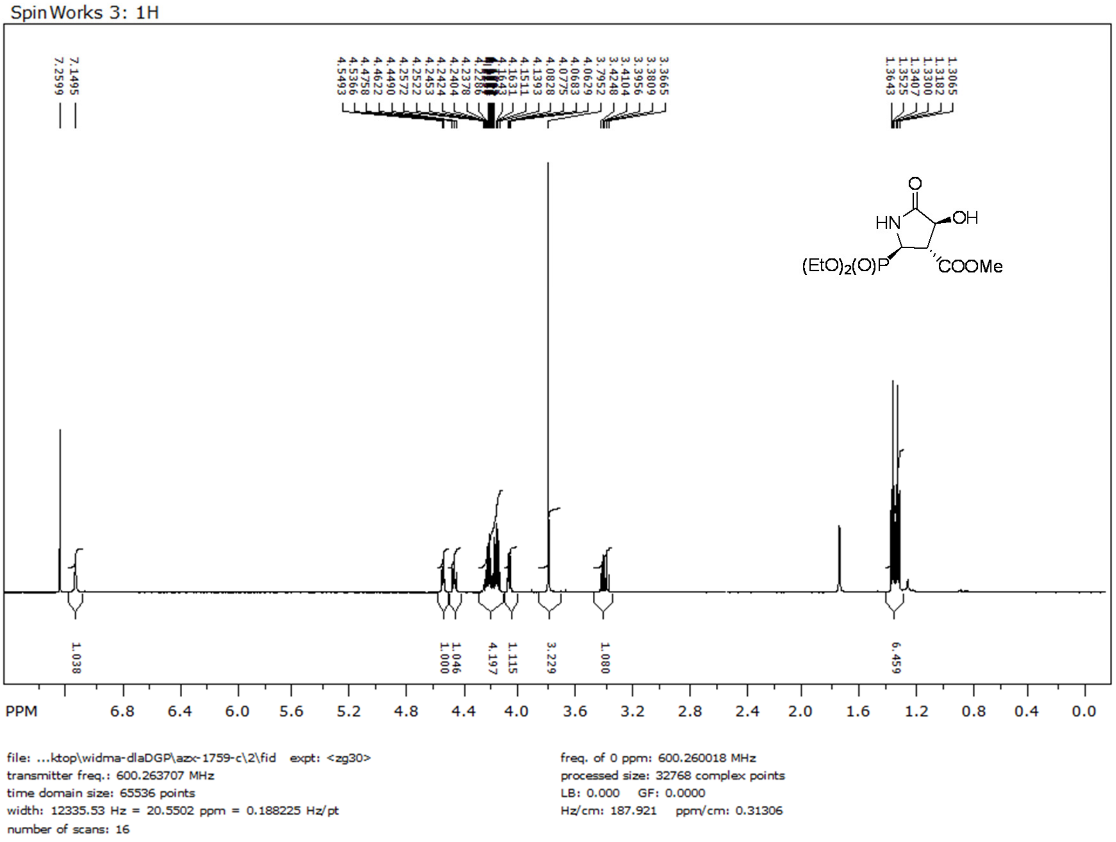

Figure S12: ^{1}H NMR Spectrum for **18** in $CDCl_3$

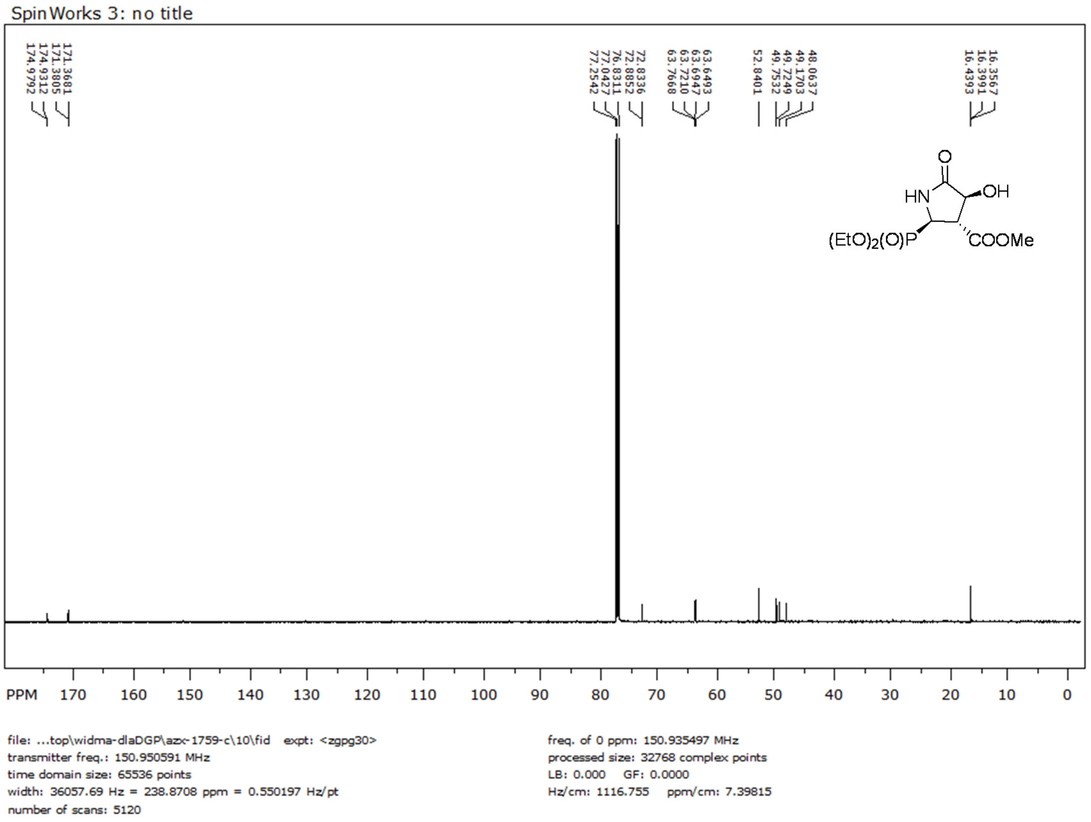

Figure S13: ^{13}C NMR Spectrum for **18** in $CDCl_3$

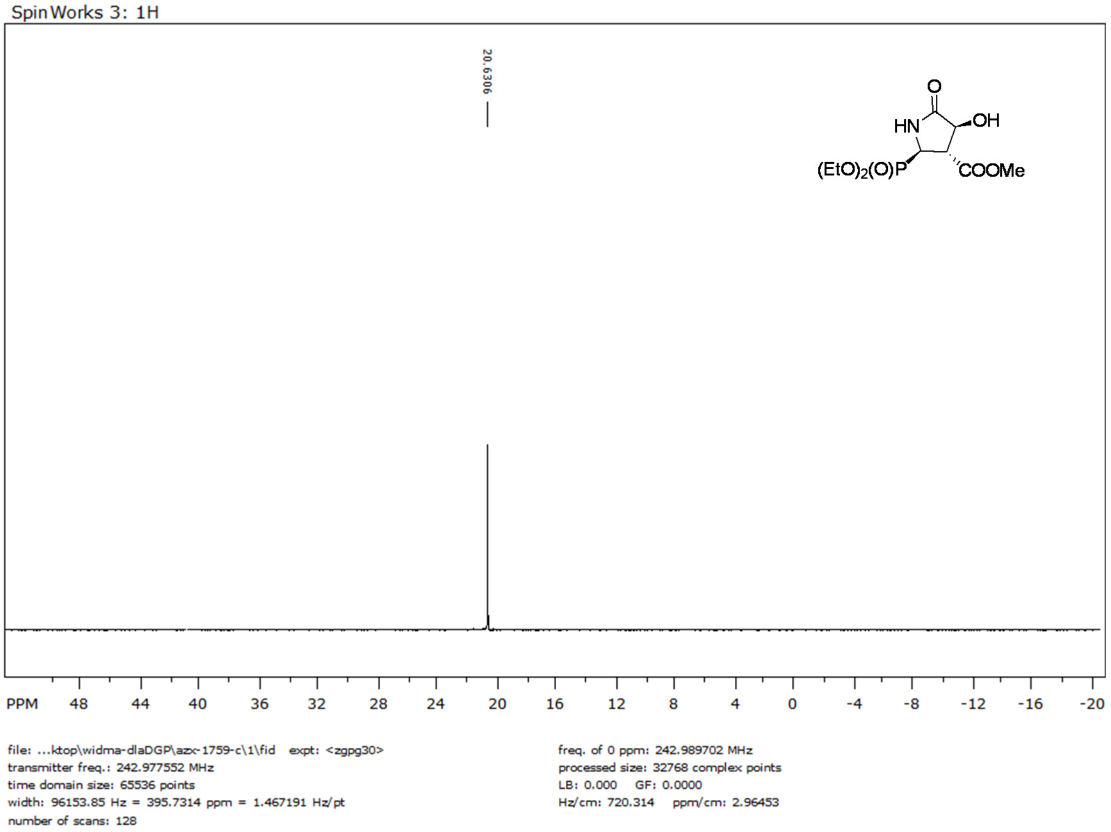

Figure S14: ^{31}P NMR Spectrum for **18** in $CDCl_3$

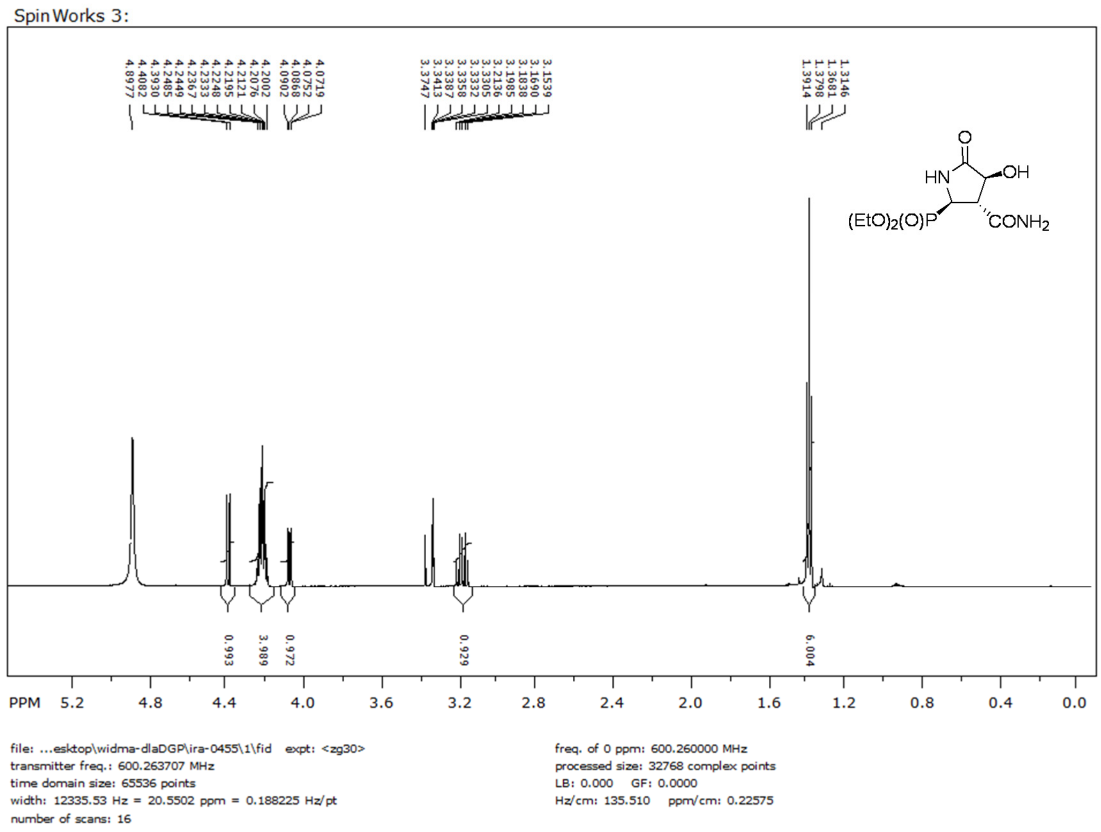

Figure S15: ^{1}H NMR Spectrum for **24** in CD_3OD

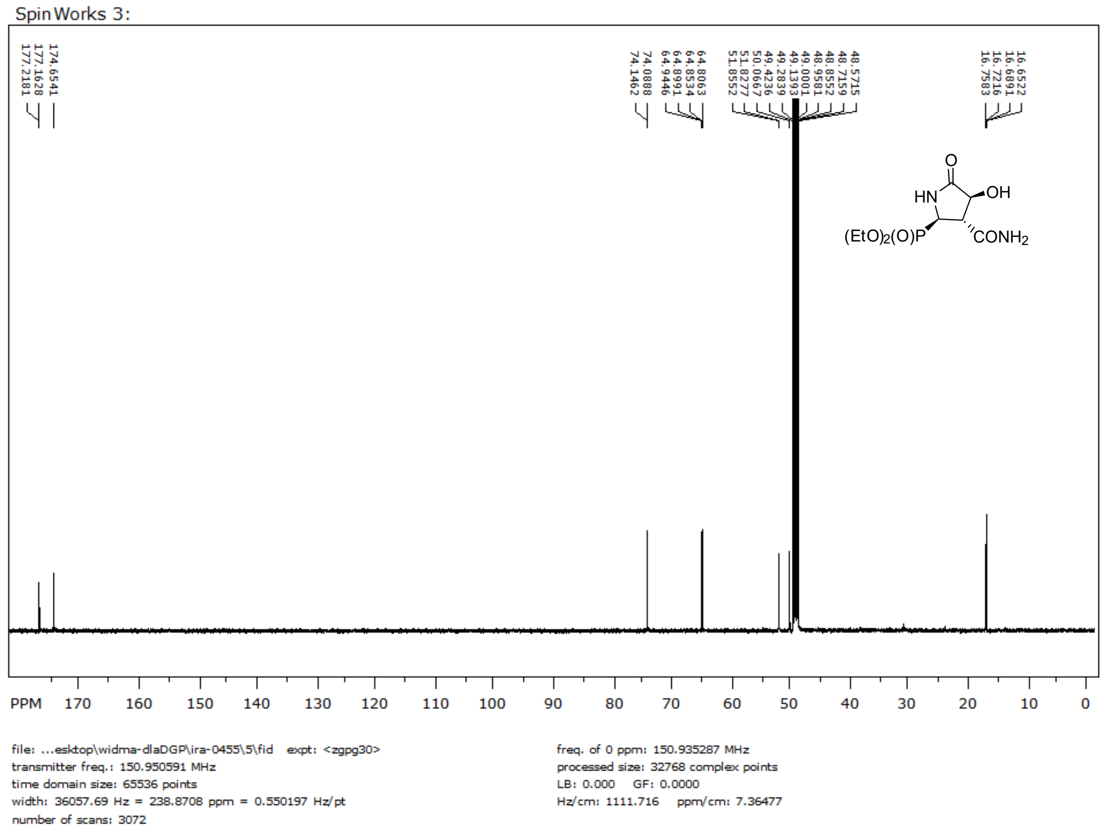

Figure S16: ^{13}C NMR Spectrum for **24** in CD_3OD

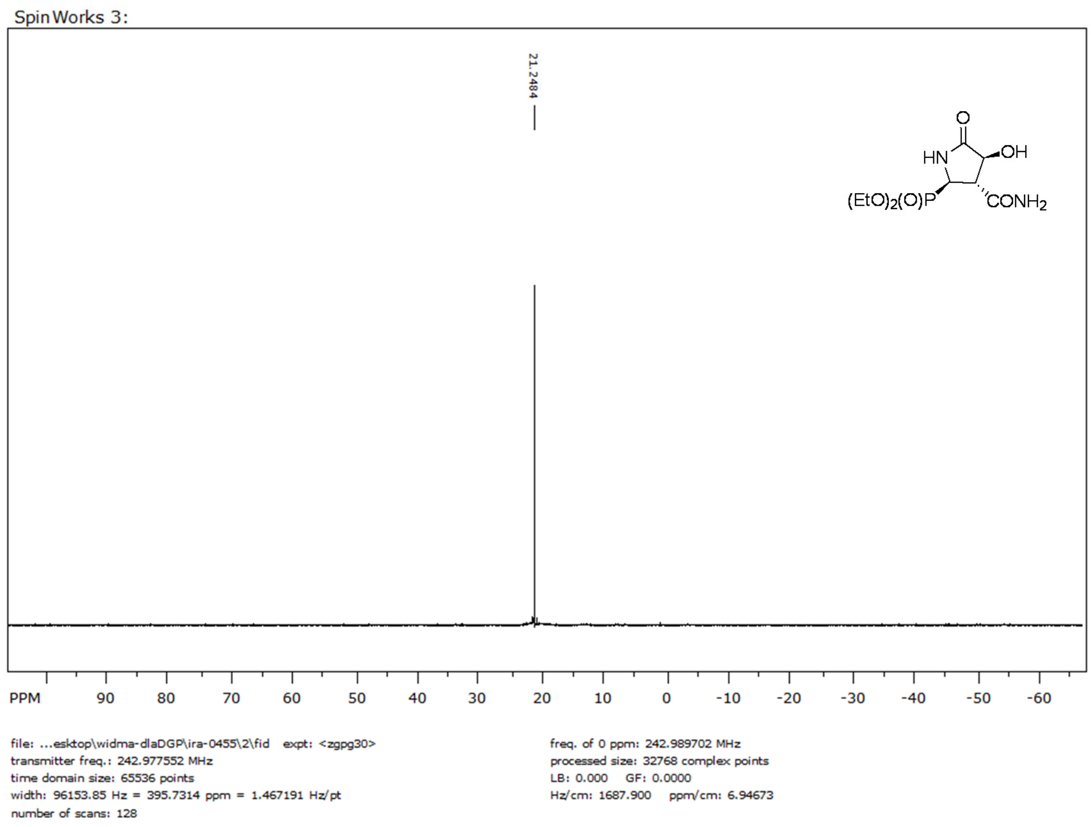

Figure S17: ^{31}P NMR Spectrum for **24** in CD_3OD

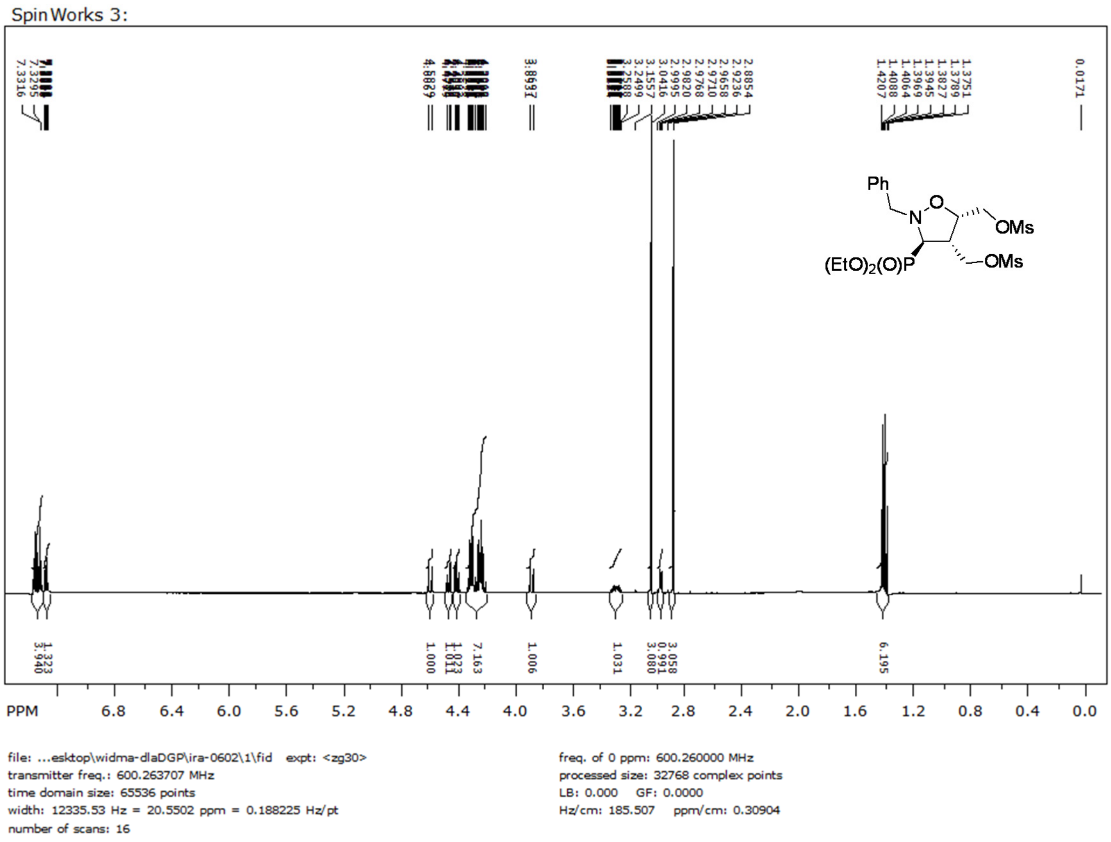

Figure S18: ^{1}H NMR Spectrum for **25** in $CDCl_3$

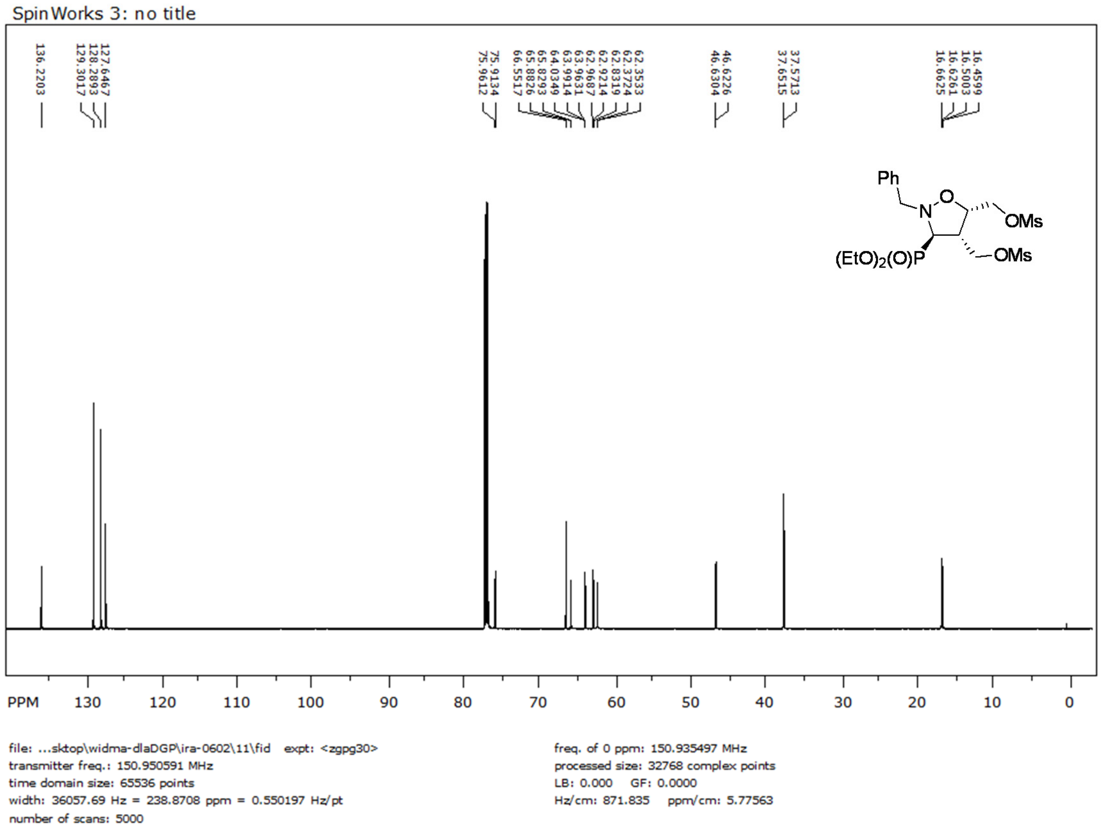

Figure S19: ^{13}C NMR Spectrum for **25** in $CDCl_3$

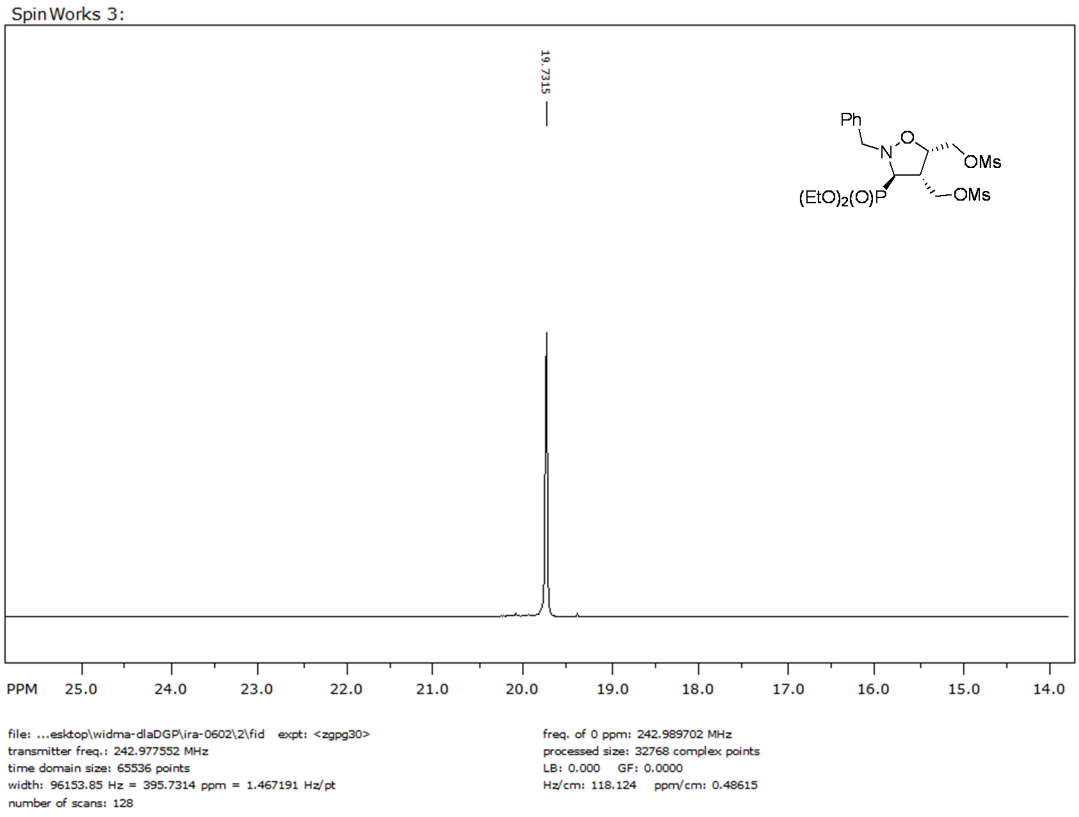

Figure S20: ^{31}P NMR Spectrum for **25** in $CDCl_3$

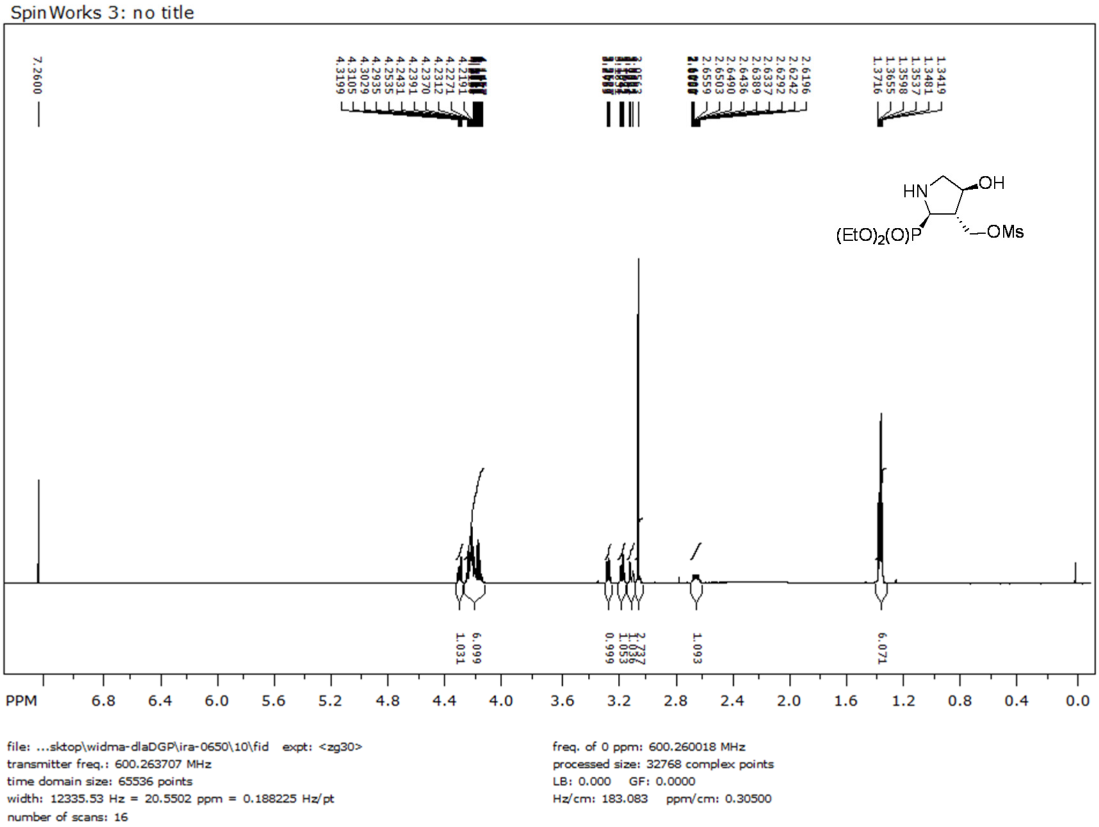

Figure S21: ^{1}H NMR Spectrum for **27** in $CDCl_3$

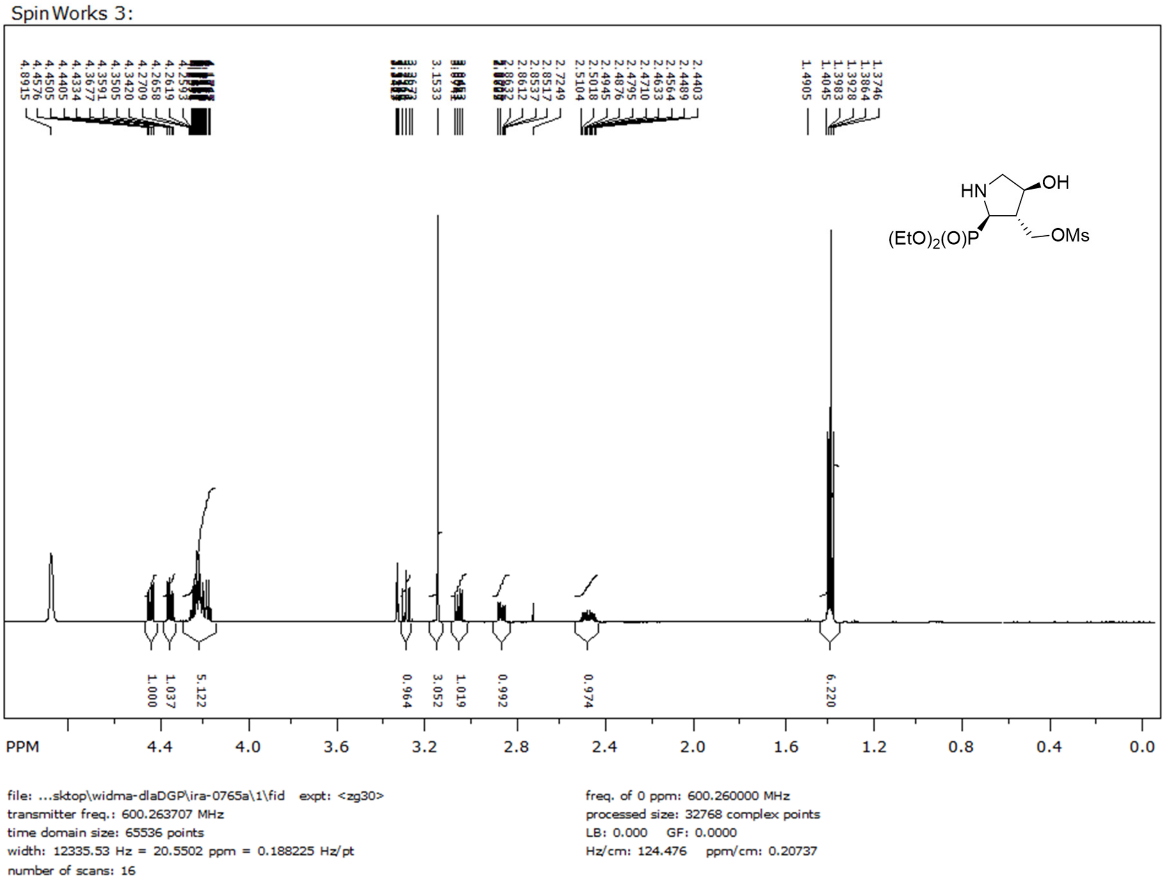

Figure S22: ^{1}H NMR Spectrum for **27** in CD_3OD

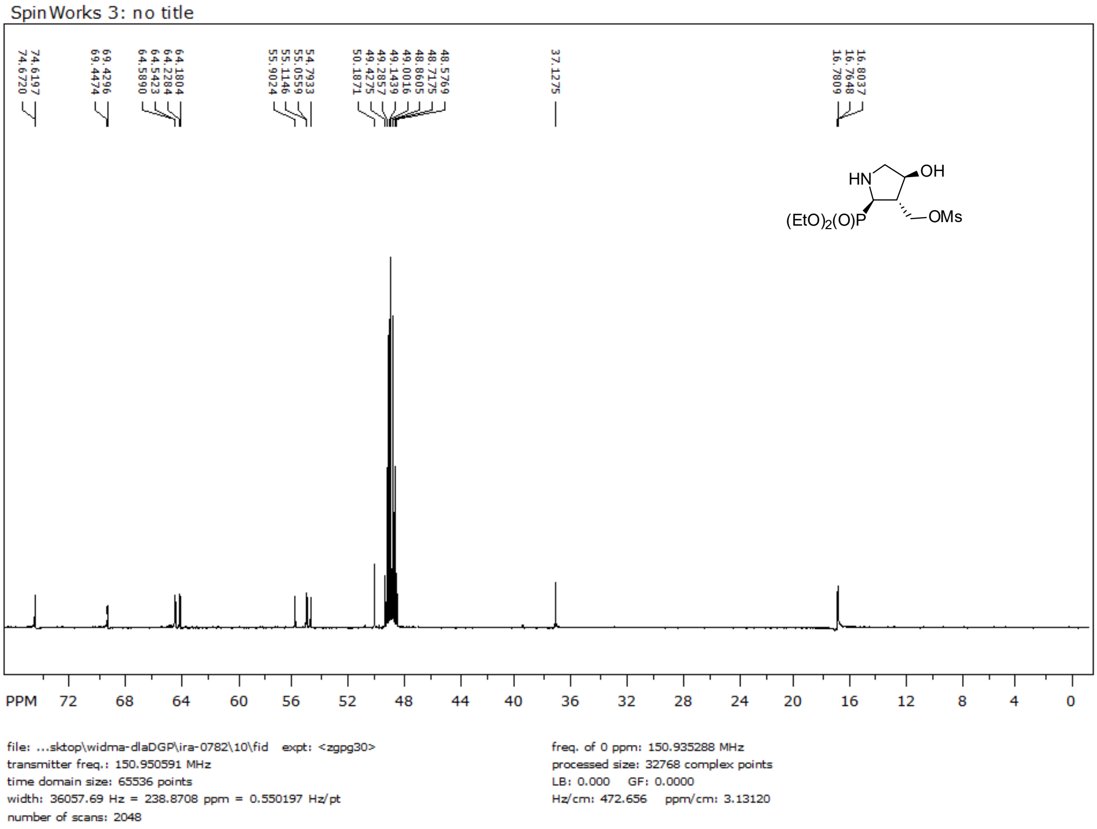

Figure S23: ^{13}C NMR Spectrum for **27** in CD_3OD

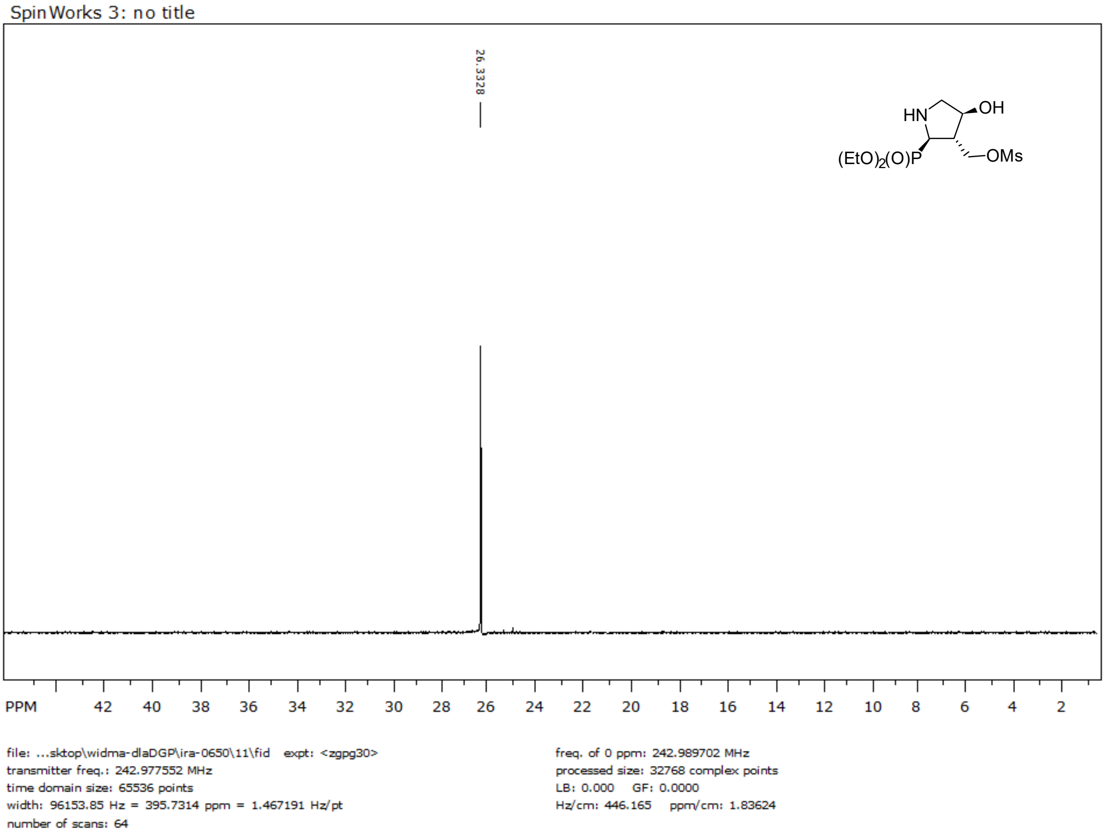

Figure S24: ^{31}P NMR Spectrum for **27** in $CDCl_3$

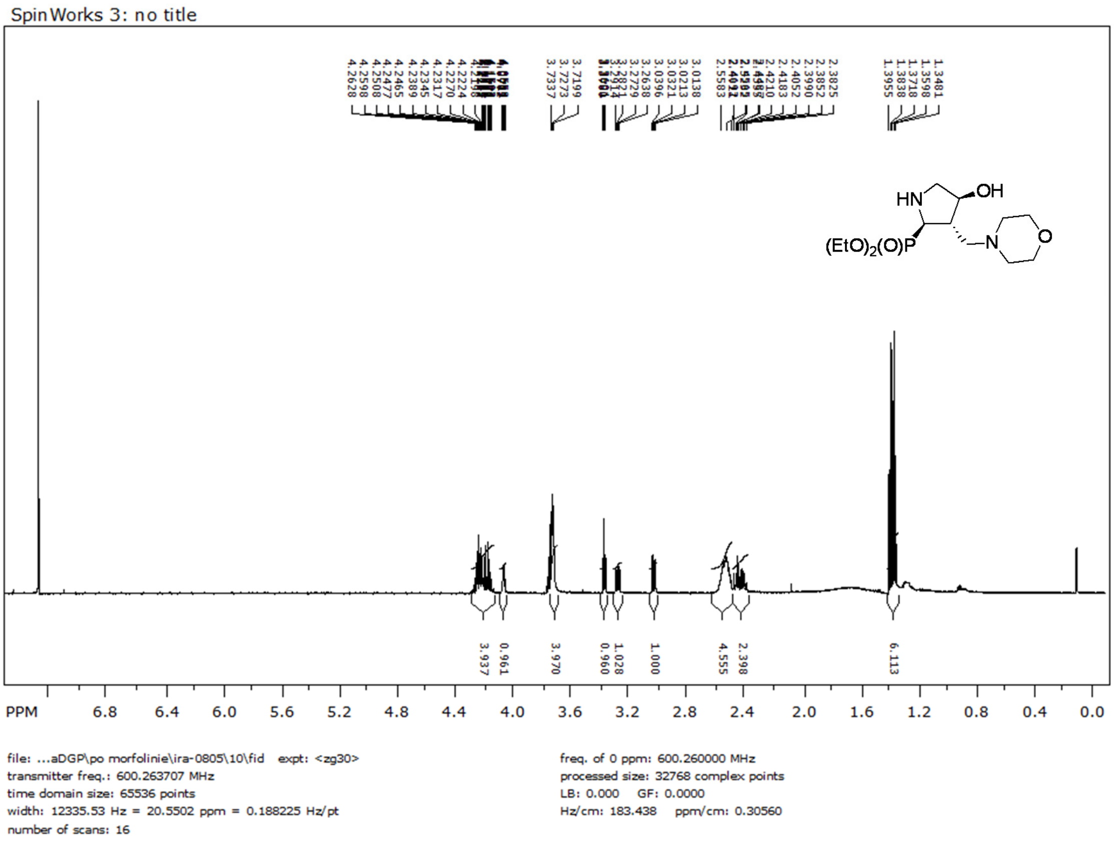

Figure S25: ^{1}H NMR Spectrum for **28** in $CDCl_3$

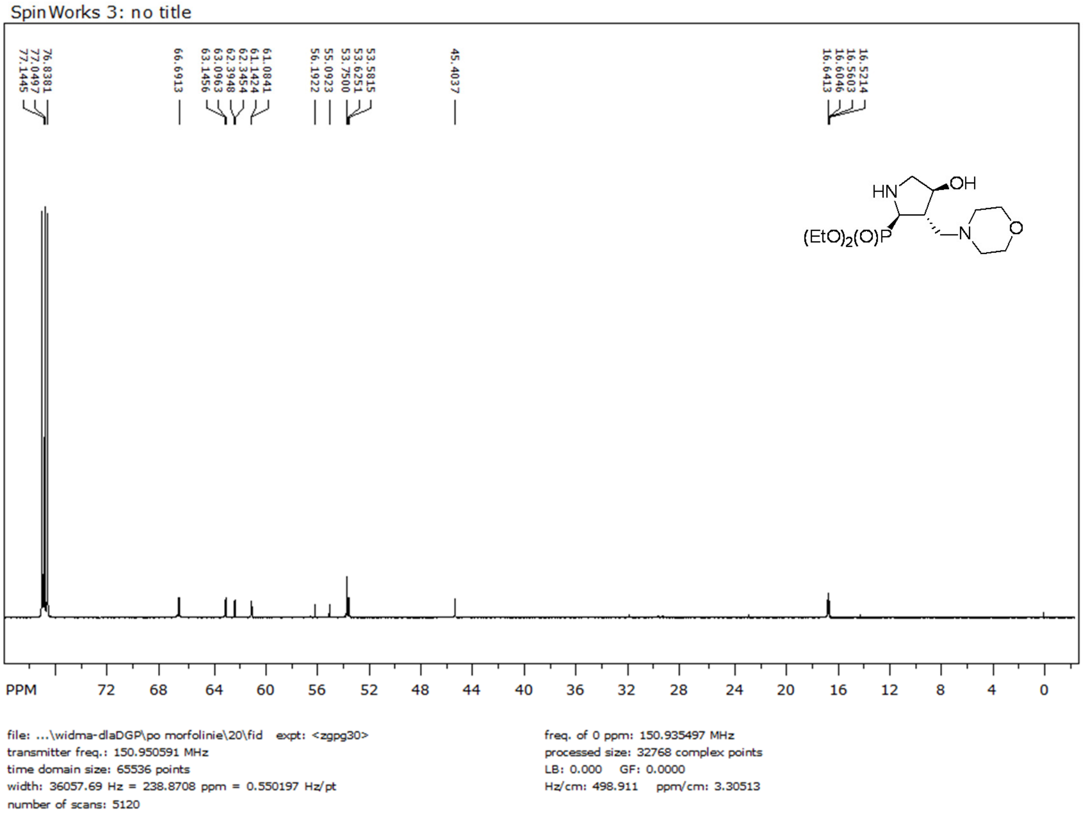

Figure S26: ^{13}C NMR Spectrum for **28** in $CDCl_3$

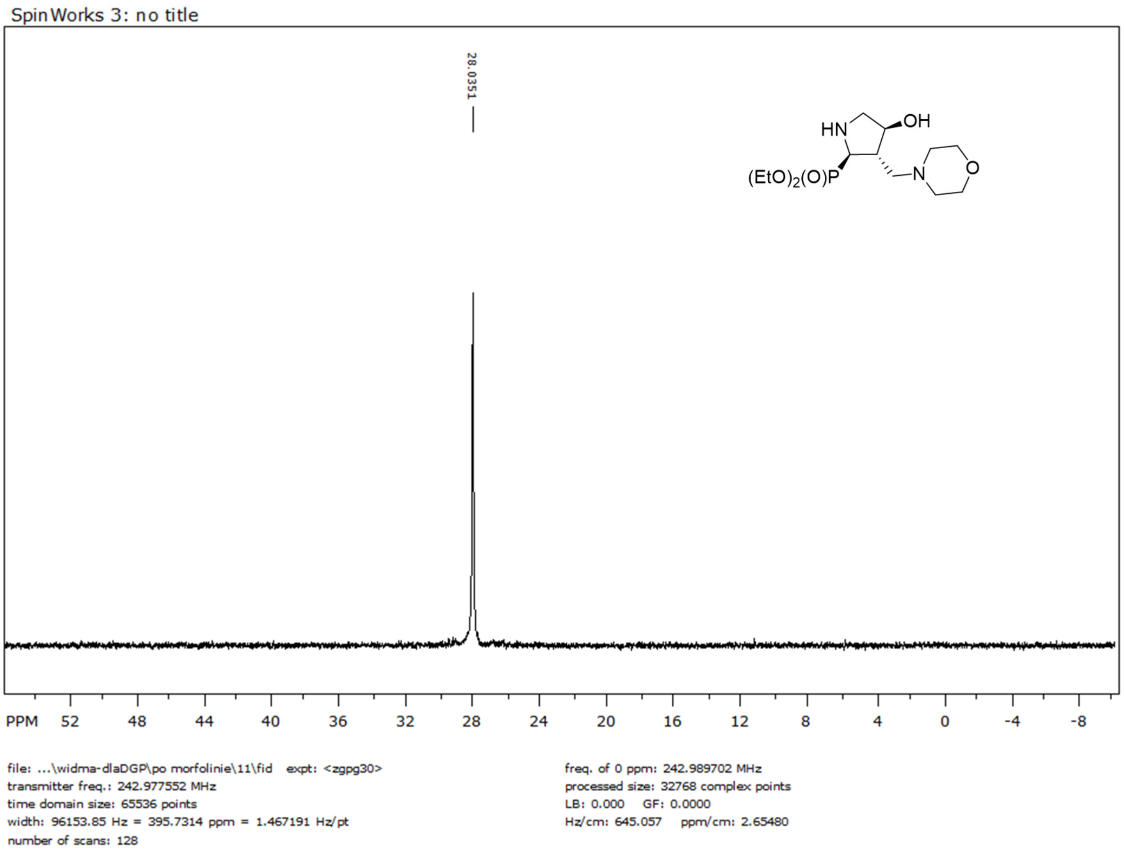

Figure S27: ^{31}P NMR Spectrum for **28** in $CDCl_3$

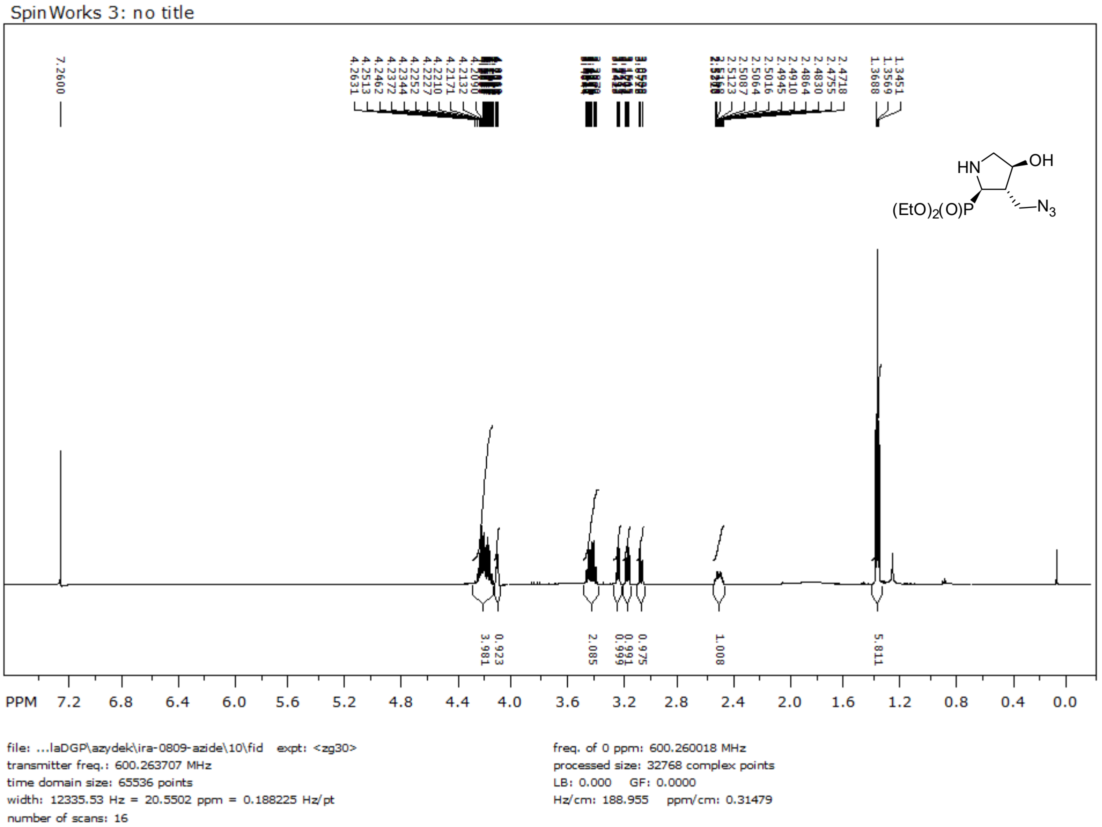

Figure S28: ^{1}H NMR Spectrum for **29** in $CDCl_3$

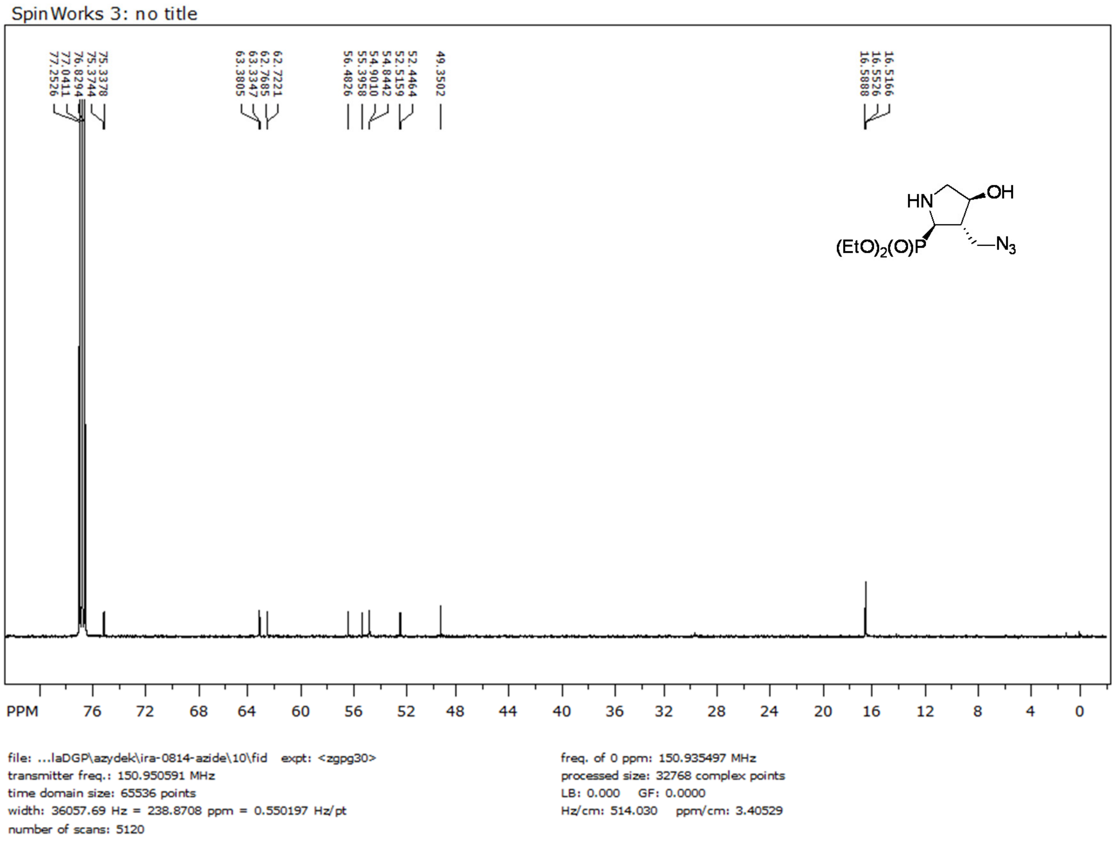

Figure S29: ^{13}C NMR Spectrum for **29** in $CDCl_3$

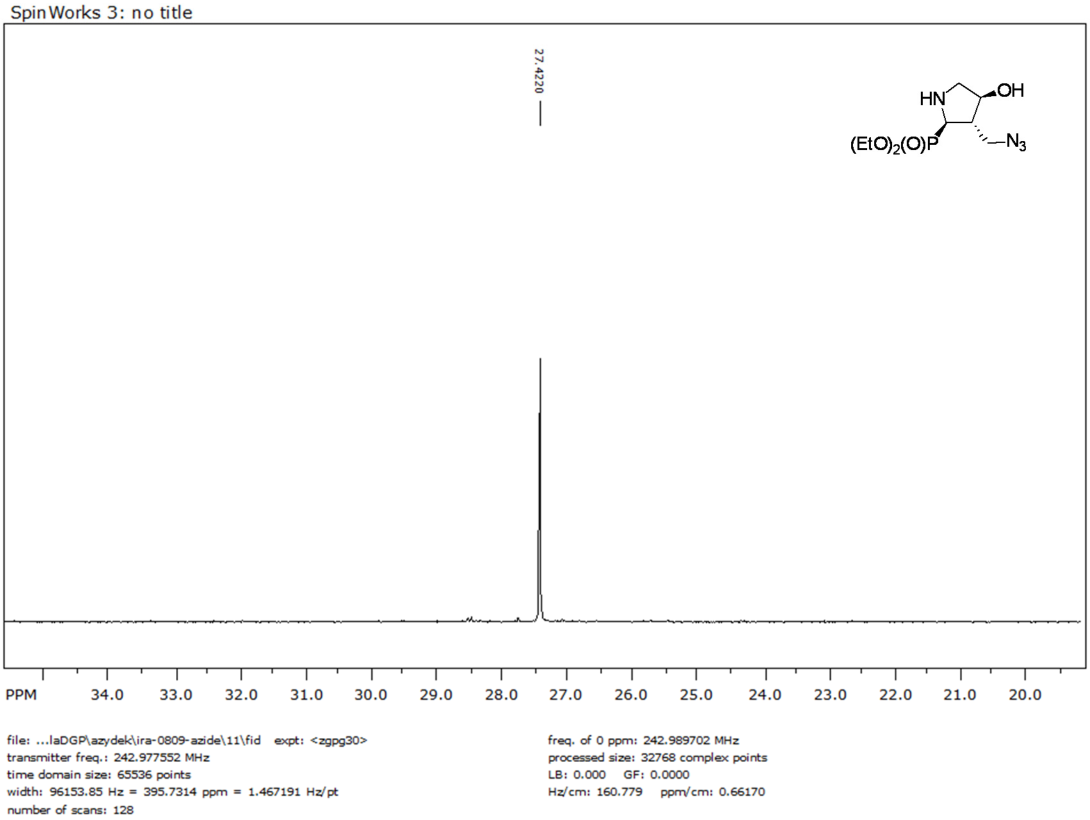

Figure S30: ^{31}P NMR Spectrum for **29** in $CDCl_3$

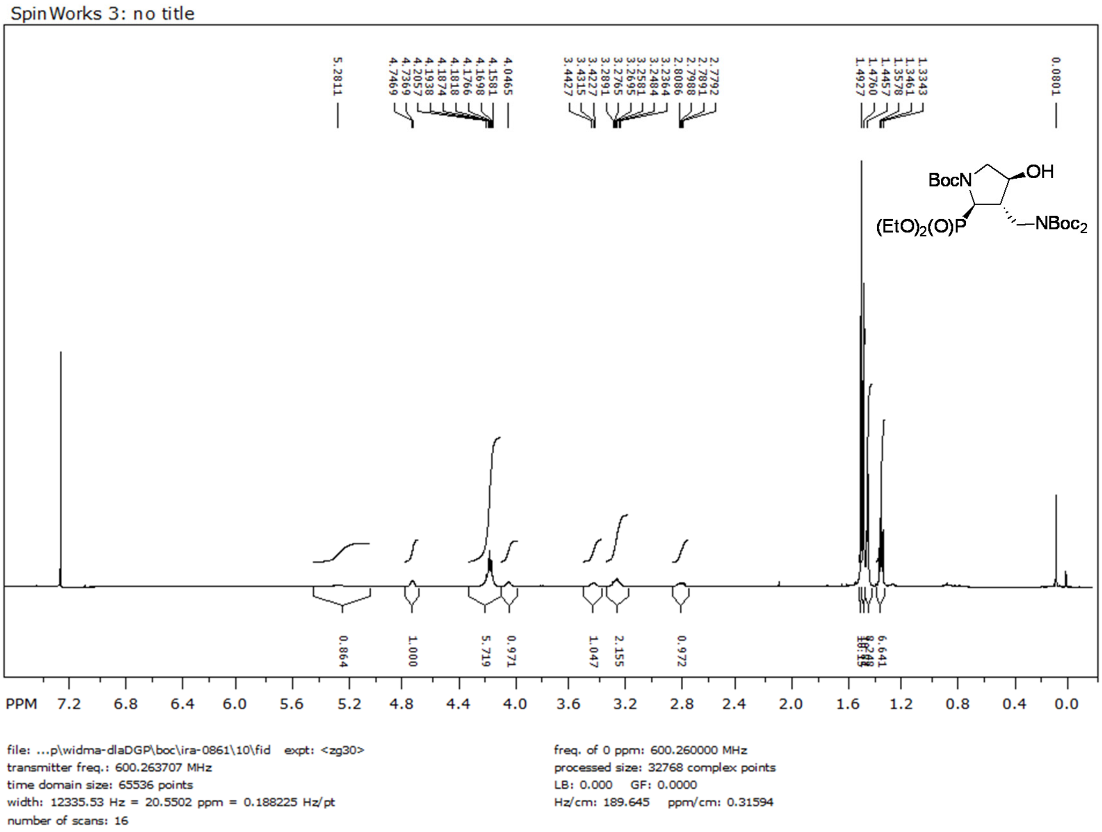

Figure S31: ^{1}H NMR Spectrum for **30** in $CDCl_3$

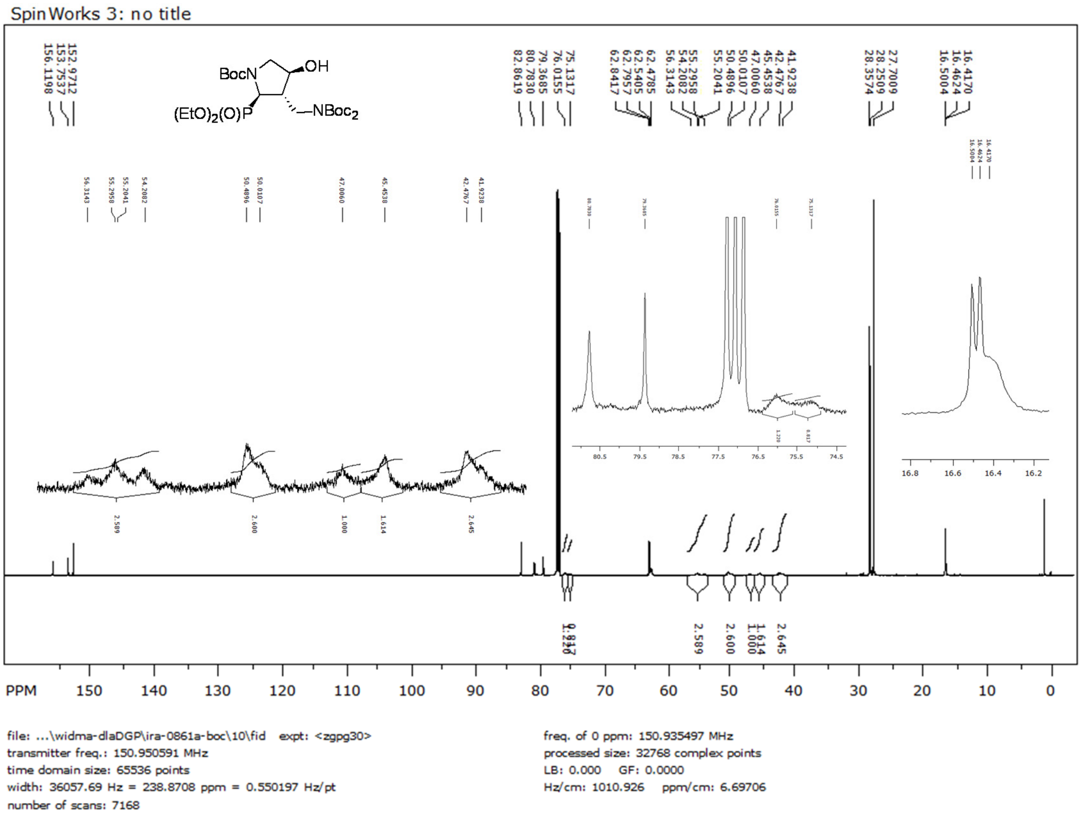

Figure S32: ^{13}C NMR Spectrum for **30** in $CDCl_3$

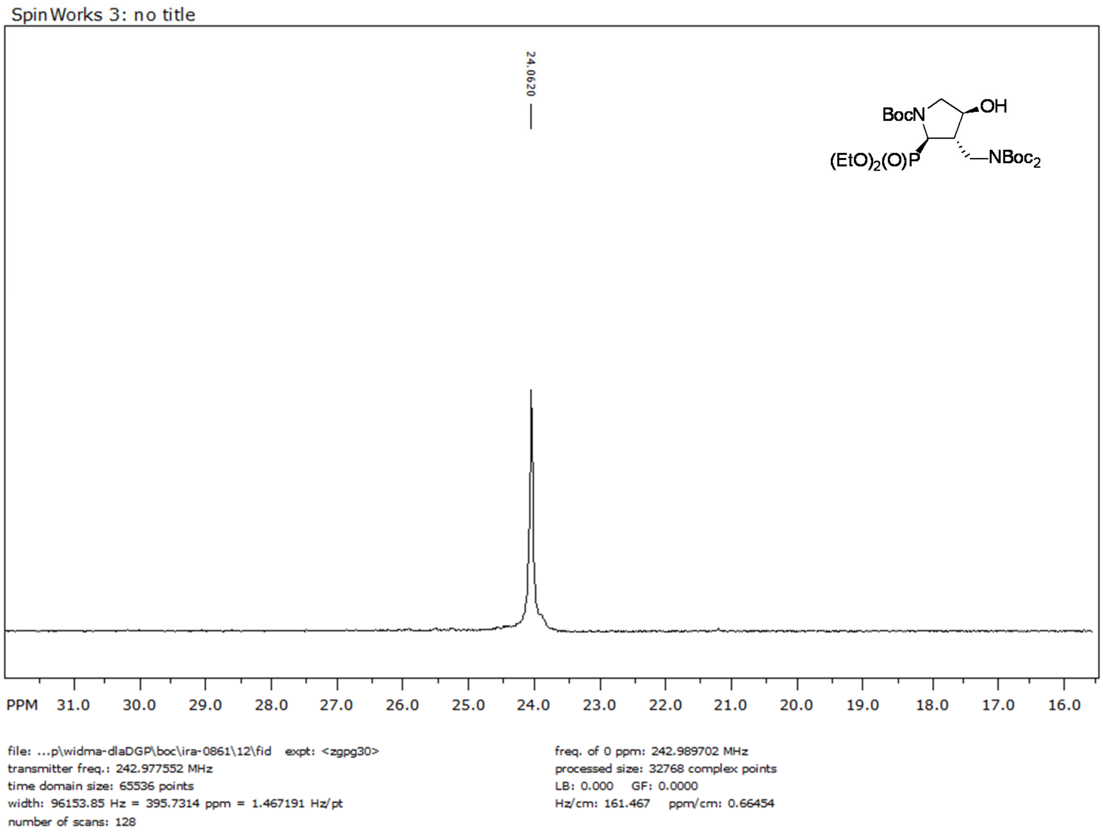

Figure S33: ^{31}P NMR Spectrum for **30** in $CDCl_3$
